# What is the next structure? Guessing enhances L2 syntactic learning in a syntactic priming task

**DOI:** 10.3389/fpsyg.2023.1188344

**Published:** 2023-06-29

**Authors:** Alaa Alzahrani

**Affiliations:** Department of English, College of Language Sciences, King Saud University, Riyadh, Saudi Arabia

**Keywords:** L2 syntactic learning, L2 prediction, L2 syntactic priming, L2 Arabic speakers, the implicit learning account

## Abstract

Previous psycholinguistic research has shown that Second Language (L2) speakers could learn from engaging in prediction. Few works have directly examined the relationship between prediction and L2 syntactic learning. Further, relatively limited attention has been paid to the effects of two linguistic factors in this area: structure type and L2 proficiency. Using a mixed experimental design, 147 L2 Arabic speakers with varying L2 proficiency levels completed two syntactic priming experiments, each targeting a different structure: (a) the dative and (b) Temporal Phrases (TP). The experimental conditions required participants to predict what the upcoming sentence’s structure would be. The experimental conditions differed in the degree of engagement in prediction error. Results suggested that Arabic L2 speakers at different proficiency levels showed enhanced priming and short-term learning for two syntactic structures (PO, fronted TP) when (a) instructed to guess only (constrained condition) as well as when (b) instructed to guess and compute the prediction error (unconstrained condition), relative to the controls. These results imply a guessing benefit for priming and short-term learning. Participants also experienced different priming effects by structure type, but there was no significant effect for proficiency. The theoretical and practical implications of these findings are discussed.

## Introduction

One important question in the psycholinguistic literature concerns the relationship between prediction and language learning. The influential implicit-learning account (Chang et al., 2006; Chang and Fitz, 2014; Dell and Chang, 2014) assumes that language speakers tacitly engage in forming predictions about the upcoming utterance and proposes that the difference between what is predicted and what is actually observed, known as the prediction error, drives learning. This has become known as error-based learning. Although the implicit learning account is a predominant framework in First Language (L1) and Second Language (L2) psycholinguistic research (for a review, Bovolenta and Marsden, 2021b), one of its key proposals remains contested, namely that prediction drives learning by the computation of prediction error (e.g., Rabagliati et al., 2016). Few works have directly examined whether prediction and prediction error promote L2 syntactic learning (Grüter et al., 2021; Bovolenta and Marsden, 2021a). This study extended prior work to examine the link between prediction and L2 syntactic learning in a syntactic priming task.

## Literature review

### Defining prediction and prediction error

Prediction here means the implicit, context-based pre-activation of upcoming linguistic information (e.g., Pickering and Gambi, 2018; Huettig et al., 2022) as well as the explicit, conscious act of guessing about the next input (Potts et al., 2019; Grüter et al., 2021). A dual view of prediction fits well with the observation that L2 prediction of syntactic information involves both automatic (e.g., unconscious) and non-automatic (e.g., conscious) processes (Ito and Pickering, 2021). Meanwhile, prediction error is typically defined as a mismatch between a predicted input and the observed input (Chang et al., 2006).

## Related theories

### The implicit learning account: the error computation proposal

One major account that connects prediction with learning is the influential implicit-learning account (Chang et al., 2006). Chang et al. (2006) accounted for the relationship between engaging in prediction and language learning using computational modeling. Based on their findings from the computational model, Chang et al. (2006) proposed that priming occurs because language users use an error-based implicit learning mechanism. When an expected structure is not observed in the actual input, the language speaker is believed to adjust or update her prediction in the direction of the input, eventually leading to learning. While prediction error may promote syntactic acquisition, it might not account for how children rapidly acquire vocabulary from the first encounter. The role of prediction errors in L1 word learning is in fact debated (e.g., Rabagliati et al., 2016; Gambi et al., 2021, 2022). This suggests that results from Chang et al.’s computational model might not directly apply to how humans learn a language (for a similar view, Kaan and Grüter, 2021).

Another important point is that implicit memory systems are believed to be the primary mechanism within this account. Although this framework may offer a satisfactory explanation for how individuals learn from tacit predictions, it does not address the issue of learning from explicit predictions. It is conceivable that implicit and explicit predictions interact with one another, but the present inquiry sought to explore the unique contribution of explicit prediction in enhancing learning. This study posits that L2 speakers could learn from explicit prediction and implicit prediction. The main aim of this study was to investigate whether explicit prediction benefits learning, as implicit prediction is presumed to do.

Furthermore, error-based learning seems to be more context-constrained than originally proposed in the implicit learning account. Evidence for this comes from a syntactic priming study which showed that L1 and L2 English speakers did not benefit from prediction errors when primed to more complex structures compared to the less complex ones (Kaan et al., 2019). Learning from prediction errors was in part modulated by the type of syntactic structure, suggesting that such learning occurs under specific contextual factors. A context-constrained view of error-based learning fits well with the utility-based approach (Kuperberg and Jaeger, 2016). This account proposes that prediction occurs only under specific contexts, and predicts that it varies across individuals (e.g., language proficiency) and within individuals (e.g., structure type). The concept of utility explains such differences in prediction. Under a utility-based approach to prediction, it is costly to predict based on unstable linguistic knowledge, and thus, less proficient L2 speakers do not find it useful to predict the next input during sentence comprehension since prediction errors would outweigh prediction success (high error rates), leading to a reduced utility of prediction. This leads to differences between individuals, with more proficient L2 learners engaging more in prediction compared to less proficient ones. Whereas the implicit learning account tends to place prediction in the heart of language acquisition without addressing within speaker-differences, the utility approach emphasizes that error-based learning is more likely to be context-dependent. To understand within-and across-speaker variation, this study examined the effects of two linguistic factors: structure type and L2 proficiency on error-based learning.

Error-based learning could be better understood when considering the role of language proficiency. One comprehensive model that discussed this topic is Hartsuiker and Bernolet’s (2015, 2017) developmental model of shared syntax. According to this framework, beginner and less proficient L2 learners are believed to rely on the lexical content (nouns, verbs) to process the primed structure. Thus, lexical overlap between the prime (“e.g., the cowboy gave the boy the book”) and target (“e.g., the cowboy gave the woman the bag”) is expected to elicit larger priming effects for learners at that stage. In contrast, more advanced learners are predicted to be less susceptible to this lexical overlap effect.

### The reward proposal

In contrast to the psycholinguistic perspective, researchers in the neuroscience field have revealed that one main determinant for successful prediction-based learning is the speakers’ state curiosity level (see Gruber and Ranganath, 2019). In this line of work, curiosity is defined as a context-modulated cognitive state that triggers information seeking to minimize uncertainty rather than a stable personal trait (e.g., van Lieshout et al., 2018). Based on neuroscientific evidence, Murayama (2022) recently proposed the reward-learning framework to explain the positive effect of curiosity on prediction-based learning and learning in general. This framework suggests that curiosity-related enhancement in memory and retention is due to the rewarding value of acquiring knowledge. After making a prediction, people become more interested in the actual answer (increased curiosity state) to know whether they generated an accurate prediction or not. When a knowledge gap is observed, the person engages in information-seeking while expecting the positive reward of acquiring the knowledge. Support for this reward proposal comes from learning studies demonstrating that people in high curiosity situations show activation of the reward network in their brains which causes improved consolidation of information encountered in such situations (see Gruber and Ranganath, 2019). However, the reward proposal remains underexplored in the L2 psycholinguistic literature.

### The utility approach

Another relevant framework for the present study is the utility approach proposed by Kuperberg and Jaeger (2016). This approach is largely a functional account of prediction in language processing that is not concerned with linking prediction to language learning. The advantage of this approach is that it expects within-and across-speaker variation in forming predictions, which is a topic of interest in this study. The utility approach hypothesizes that prediction is not a necessary component to language comprehension but rather an efficient tool that can aid comprehension in some contexts. It is more likely that language users will maximize the utility of prediction (use it under specific contexts) since they have limited cognitive resources that get used up under adverse conditions, making it difficult to anticipate what is coming next (e.g., Ito et al., 2018; Chun et al., 2021). Under this approach, the pre-activation of anticipated input is directly influenced by the expected utility of prediction, i.e., whether its advantages outweigh its disadvantages. This framework provides a utility-based rationale for the idea that prediction varies between groups and within individuals. The utility of prediction is assumed to vary between and within individuals, and this variation depends on the task, the individual’s goal, and the stimuli-structure. Overall, the utility approach offers the present study a suitable ground to investigate how different factors (structure type and L2 proficiency) influence between-group and within-individual variation in prediction-based learning.”

To sum up, the three accounts offer complementary explanations for how prediction could support learning. The implicit learning account proposes an implicit learning mechanism through which language users learn the primed structure by adjusting knowledge to minimize prediction errors. A core idea in this account is that speakers unconsciously learn from predictions. On the other hand, the reward framework posits that people form predictions using both implicit and explicit memory systems and they change their knowledge to satisfy the internal reward system. Thus, in contrast to the implicit learning account, the reward framework expects that people can acquire knowledge consciously and unconsciously following making a prediction. Unlike the previous two accounts, the utility approach puts more emphasis on the factors mediating prediction rather than prediction-based learning. This approach highlights that engagement in prediction is variable among language speakers and suggests that across (e.g., L2 proficiency) and within-individual factors (e.g., type of primed structure) play a key role in triggering or limiting the formation of predictions.

### Prediction in L1 and L2 language learning

Several L1 and L2 studies have examined whether experiencing prediction errors trigger vocabulary and syntactic learning. Two forms of the prediction error hypothesis exist in the literature (Rabagliati et al., 2016) as proposed by the implicit learning account (Chang et al., 2006). Under a strong form, prediction is said to cause learning via the computation of prediction error (see Gambi, 2021). A less committed approach posits that prediction errors aid learning (e.g., Bovolenta and Marsden, 2021b). L1 word learning studies have provided mixed findings for the strong version of the hypothesis (Reuter et al., 2019; Fazekas et al., 2020; Gambi et al., 2021, 2022). The weaker form of the prediction error hypothesis is typically investigated in L2 research (see Bovolenta and Marsden, 2021b). Two L2 studies have explored the effect of guessing on vocabulary learning and reported different results (Potts et al., 2019; Wang et al., 2022). Generating incorrect guesses for the meaning of L2 idioms led to difficulties in recalling the correct idiom meanings one week later (Wang et al., 2022), but this was not observed for learning L2 words (Potts et al., 2019).

Two other studies have directly investigated the relationship between prediction error and L2 syntactic learning and both found a positive effect (Grüter et al., 2021; Bovolenta and Marsden, 2021a). For example, Grüter et al. (2021) used a syntactic priming paradigm and asked L2 English-speaking participants to predict the structure of an upcoming sentence (prime) and then required them to compare their prediction and the actual sentence structure (experimental condition). Participants in the experimental condition showed larger syntactic priming effects compared to the control who were not asked to predict. However, an issue in the experimental condition makes it hard to interpret the findings from this study. In the experimental condition, participants had to (a) guess and type in a picture description and then were asked to (b) indicate whether their guesses matched the actual picture description. The observed priming effects in this condition could be due to (a) increased curiosity following making a guess (i.e., wanting to know whether the guess was correct or not) not because of (b) engaging in comparison (i.e., computation of prediction error). An improved design is needed to separately measure the two mechanisms to better understand how prediction could benefit L2 syntactic learning in a syntactic priming task.

To sum up, there are conflicting findings for prediction error on L2 vocabulary acquisition, but there is some support for its role on L2 syntactic learning. Due to the limited number of studies examining the links between prediction and L2 language learning, some investigators have called for further research on this topic (Bovolenta and Marsden, 2021b).

### Prediction in syntactic priming

A well-studied psycholinguistic finding is that language users tend to reuse a syntactic structure that has been recently encountered or produced (Mahowald et al., 2016). The tendency to repeat a previously experienced structure is known as syntactic priming or structural priming (Bock, 1986; Pickering and Ferreira, 2008). Evidence for the role of prediction error in syntactic priming and learning comes from two known effects in the L1 and L2 syntactic priming literature: the inverse frequency effect and the verb surprisal effect (Jaeger and Snider, 2008; Kaschak et al., 2011; Fine et al., 2013; Fine and Jaeger, 2013; Jaeger and Snider, 2013; Peter et al., 2015; Kaan and Chun, 2017, 2018; Dempsey et al., 2020; Fazekas et al., 2020; Montero-Melis and Jaeger, 2020; Grüter et al., 2021; Yang et al., 2021; Chen et al., 2022).

Several syntactic priming models have been proposed to account for the observed priming effects (Pickering and Branigan, 1998; Chang et al., 2006; Hartsuiker et al., 2008; Reitter et al., 2011; Segaert et al., 2016; Heyselaar et al., 2021). Relevant to this work is Reitter et al.’s (2011) account which proposes that durable priming occurs due to a learning mechanism that changes the base-activation of abstract structures in long-term memory. In this view, each syntactic structure has a specific base-level activation in speakers’ long-term memory, indicating the total number of retrievals for that structure. Priming increases the base-level activation of the syntactic structure in long-term memory, which in turn boosts immediate and future production of that structure, resulting in short-term and long-term priming, respectively. While the implicit learning account assumes error-driven learning underlies syntactic priming (Chang et al., 2006), Reitter et al. (2011) posits that priming arises from base-level learning in addition to spreading activation. This model explains long-term syntactic priming (after trials, sessions, days, weeks) via base-level learning without recourse to error-driven learning. A weight-adjusting learning mechanism is responsible for changing the base-level activation of abstract combinatorial nodes in the long-term memory each time a syntactic structure is encountered. Base-level learning occurs through extensive (frequent) and recent exposure/retrieval. A participant who has frequently and recently processed a structure in a syntactic priming task is likely to have a higher base-level activation for that structure in long-term memory, leading to an observable syntactic priming effect. Note, however, that base-level activation is thought to decay over time in case of limited exposure.

## Factors mediating prediction-based learning

### Structure type

Four studies so far have directly investigated the influence of structure type on syntactic priming. Two of these were concerned with sentence comprehension (Kaan et al., 2019; Chun et al., 2021), and the other two with sentence production (Shin and Christianson, 2012; Coumel, 2021). The comprehension studies revealed that learning from prediction errors could be challenging for more complex structures, suggesting that prediction-based learning might not be suitable for all syntactic structures. However, these studies examined syntactically ambiguous structures (garden path sentences, ambiguous relative clauses) which makes it difficult to separate syntactic ambiguity effects from syntactic complexity effects. The production studies reported contrasting results. One study (Shin and Christianson, 2012) found that the less complex structure (phrasal verbs) had larger priming effects than the more complex structure (double object datives). The other study (Coumel, 2021) reported larger immediate priming and long-term learning for the more complex structure (passives) compared to the less complex one (fronted temporal phrases). Yet, the production priming studies did not explore the effect of explicit prediction across structure types.

### L2 proficiency

In the predictive processing literature, existing evidence suggests that L2 proficiency is more likely to exhibit a marginal effect (Mitsugi and Macwhinney, 2016; Perdomo and Kaan, 2019) than a significant effect (Hopp, 2016). In syntactic priming research, the effect of L2 proficiency has been examined using largely intermediate speakers (Grüter et al., 2021), largely advanced speakers (Coumel, 2021), or both (Ruf, 2011; Montero-Melis and Jaeger, 2020). Studies that investigated L2 speakers from different proficiency levels reported that there were increasing priming effects with increasing proficiency. However, only one of these studies has examined the proficiency effect in relation to explicit prediction (Grüter et al., 2021). Further research is needed to examine the differential effects of L2 proficiency on prediction-based learning.

### The present study

Using the syntactic priming paradigm, this study aims to examine prediction-based learning across two Modern Standard Arabic (MSA) syntactic structures among Arabic L2 speakers from different proficiency levels. Two goals motivate this examination. One is to understand how prediction supports L2 syntactic learning via a syntactic priming task. To do so, this study adapted the design of Grüter et al.’s (2021) to closely examine how engagement in prediction error and in explicit prediction (Q1) could contribute to L2 syntactic priming and learning (Chang et al., 2006). The second goal is to assess the effects of two linguistic factors (structure type and L2 proficiency; Q2 and Q3) to understand within and across-speaker variation in prediction-based learning (Kuperberg and Jaeger, 2016). The aims guided the following research questions:

1. Is there an effect of condition on prediction-based learning via a syntactic priming task?
2. To what extent prediction-based learning via a syntactic priming task is affected by the type of prime structure?
3. To what extent prediction-based learning via a syntactic priming task is affected by L2 proficiency level?

## Methods

### Participants

The participants were 147 L2 speakers of Arabic (Female speakers = 53) who were assigned randomly to one of the three conditions. Additional six participants were excluded from the analysis due to a technical issue (*n* = 1), not completing the delayed session (*n* = 2), producing only “other” responses (explained below) across all phases in the first session (*n* = 2), completing the delayed session more than 16 days after the priming task (*n* = 1). All L2 participants were either current or previous students at the Arabic linguistics institute at King Saud University. Previous students had to be currently enrolled in a program at the university to be eligible for participation. The participants came from different L1 backgrounds (*N* = 40) due to the limited number of L2 speakers who share the same L1 at the recruitment site. All participants provided informed consent online before starting the study and received financial compensation upon task completion. The study was approved by the Humanities and Social Sciences Research Ethics Committee at King Saud University under number/KSU-HE-22-271.

The participants’ demographic information is presented in Supplementary Table S1, along with the results of between-group comparisons. The Kruskal-Wallis test revealed no significant differences between the conditions in any of the variables in Supplementary Table S1. The participants rated their proficiency in (a) speaking, (b) understanding spoken language, as well as (c) reading, and the average of these ratings was computed and reported (Coumel et al., 2022).

### Target structures

Two MSA target structures were investigated: the dative alternation and temporal phrases (TP). Each structure has two alternations that deliver the same message (Supplementary Table S2). Both structures are well-studied in the related literature, suggesting that they constitute appropriate testing ground for the syntactic priming effect (Ruf, 2011; Mahowald et al., 2016; Jackson and Ruf, 2017, 2018; Jackson and Hopp, 2020; Coumel, 2021). A further motivation for the inclusion of these two structures is that they differ in their constituent structure. In Arabic, TPs contain fewer constituents compared to datives. Supplementary Figure S1 illustrates the constituents of a DO dative sentence (example 1), which starts with a verb followed by three Noun Phrase (NP) arguments. The first NP (NP1) represents the agent, the second NP (NP2) represents the recipient, and the third NP (NP3) represents the theme. Meanwhile, the Arabic TP structure is composed of a preposition and one obligatory NP. This could suggest that formulating an appropriate dative sentence might be more challenging for L2 Arabic learners as it requires the arrangement of three NPs (Al-Jadani, 2016) compared to constructing a TP structure. Examining two distinct structures could provide valuable insights into the role of prediction in syntactic priming and learning across structures.

### Design

A mixed experimental design was used, which combined a between-subject design and a within-subject design. Two syntactic priming experiments were run. Each experiment investigated one target syntactic structure (experiment 1 = datives, experiment 2 = TPs), and both followed the pretest, immediate posttest, and delayed posttest design.

### Syntactic priming task

A visual comprehension-to-production syntactic priming task was used, closely following Grüter et al. (2021). The online experiment builder Gorilla.sc (Anwyl-Irvine et al., 2020) was used to create and administer the priming task. There were three priming conditions: constrained, unconstrained, and control (Supplementary Figure S1). The unconstrained prediction and control were replicated from Grüter et al. (2021). However, an additional “constrained” condition was included in the present study to better assess the learning benefits of engaging in error computation. The constrained and unconstrained conditions differed only in the degree of engagement in error computation. Engagement in error computation was manipulated by (a) the type of information offered, (b) the type of instruction provided, and by (c) the time limit. Specifically, unlike the constrained condition, the unconstrained condition allowed participants to read their predicted sentence description and instructed them to compare it with the actual sentence description. Across both prediction conditions, participants had to guess the structure of the prime and then, in the subsequent screen, were shown the actual answer (error computation screen). In the error computation screen for the unconstrained condition, participants were shown their prediction and the actual answer, and were instructed to compare their guesses with the actual answer without imposing a time limit on this screen (self-paced progress). In the error computation screen for the constrained condition, participants were only shown the actual answer for 3,500 ms (controlled progress). A 3,500 ms time limit was imposed as this is the minimum amount of time needed to naturally articulate the Arabic sentences in the task.

A picture-sentence matching task was designed to validate the enforced time restriction in the constrained condition. Results from this task indicated that sentence comprehension was to some extent negatively impacted when the L2 Arabic speakers (*N* = 9) had only 3,500 ms to process the sentence compared to 7,000 ms (*β* = −1.31, 95% CI [−2.59, −0.03], *SE* = 0.66, *z* = −2.00, *p* = 0.045).

## Materials

### Sentence stimuli

Guidelines from previous priming research were followed to construct the sentence stimuli. Following Grüter et al. (2021), the constructed experimental sentences included only the vocabulary found in the textbooks corpus to ensure participants’ familiarity with the sentences. A corpus of all the verbs (*N* = 1,405), nouns (*N* = 1,419) and adjectives (*N* = 60) used in the participants’ textbooks was created by retrieving the vocabulary lists provided at the end of each book. Twenty-five experimental sentences were created per experiment to ensure the inclusion of an equal number of sentences across phases (baseline = 3, priming = 16, immediate posttest = 3, delayed posttest =3). No verbs or any lexical items were shared between the experimental sentences in the same phase to eliminate lexical overlap effects. Additionally, 31 fillers were constructed per experiment. Fillers across experiments included transitive and intransitive verbs that differed from those used in the experimental sentences. Almost all fillers had animate subjects to mimic the experimental sentences and minimize pattern recognition (Grüter et al., 2021; Coumel, 2021). All stimuli and R codes are available at: https://osf.io/f6rj3/?view_only=0d662971e40b4db3bb2128cf0d0ad9ff.

The acceptability of sentence stimuli was assessed in a magnitude estimation task (e.g., Jaeger and Snider, 2013). Five native Arabic speakers with MA/PhD degrees in Arabic linguistics (Female = 4) completed the task after getting their informed consent. Acceptability ratings were log-transformed and fitted using a robust linear-mixed effect model using the package robustlmm (Koller, 2016). A post-hoc pairwise comparison test using Tukey method revealed no statistically significant differences in judgment ratings between the experimental structures (fronted and non-fronted TP, PO, and DO).

Four counterbalanced lists were created that alternated between PO and DO for adjacent prime-target pairs, with each experimental pair being followed by one or two filler pairs. The same counterbalance was done for the TP structure. Placement of images representing TPs was also counterbalanced within phase. Half of the 25 images representing the TPs were placed in the upper left-hand corner of the picture, while the other half was placed in the upper right-hand corner (Jackson and Ruf, 2018; Jackson and Hopp, 2020; Coumel, 2021). Each participant saw only one list per structure.

### Picture and audio stimuli

All pictures in the priming task were labeled with the appropriate vocabulary, with the infinitive form of the verb included in bold below each picture to limit the production of unrelated structures (Branigan and Gibb, 2018). All prime sentences were also voice-recorded by a female native Arabic speaker who was instructed to use Modern Standard Arabic (MSA) and to speak as naturally as possible. Presenting prime sentences in two modalities: auditory and written was done to accommodate the L2 participants’ varying proficiency levels (Jackson and Ruf, 2017; Jackson and Hopp, 2020).

### L2 proficiency

L2 proficiency was estimated using two measures: a reading test and a self-reported questionnaire. A multiple-choice Arabic reading proficiency test was adopted from the Arabic Linguistics Institute at King Saud University. The test has 20 items and provides four possible choices for each item. The reading test was used as a proxy for Arabic proficiency due to the limited availability of standardized proficiency tests for L2 Arabic speakers (Mohamed, 2016), leading some psycholinguistic researchers to rely on Arabic program placement levels (Alhawary, 2019; Al Masaeed et al., 2020) or self-ratings (Foote et al., 2020) to measure L2 Arabic proficiency. Supplementary Figure S3 shows the scores of the Arabic reading test. This study also adopted a self-reported proficiency measure from the Arabic version of the Language Experience and Proficiency Questionnaire (LEAP-Q) (Marian et al., 2007, 2020). The LEAP-Q requires L2 speakers to rate their proficiency on (a) speaking Arabic, (b) understanding spoken Arabic, and (c) reading Arabic on a scale from 0 (minimum score) to 10 (maximum score). The self-reported Arabic proficiency ratings and the Arabic reading test scores moderately correlated, *r* (145) = 0.34, 95% CI [0.19, 0.48], *p* < 0.001 (Plonsky and Oswald, 2014).

### Procedure

**Figure 1:**
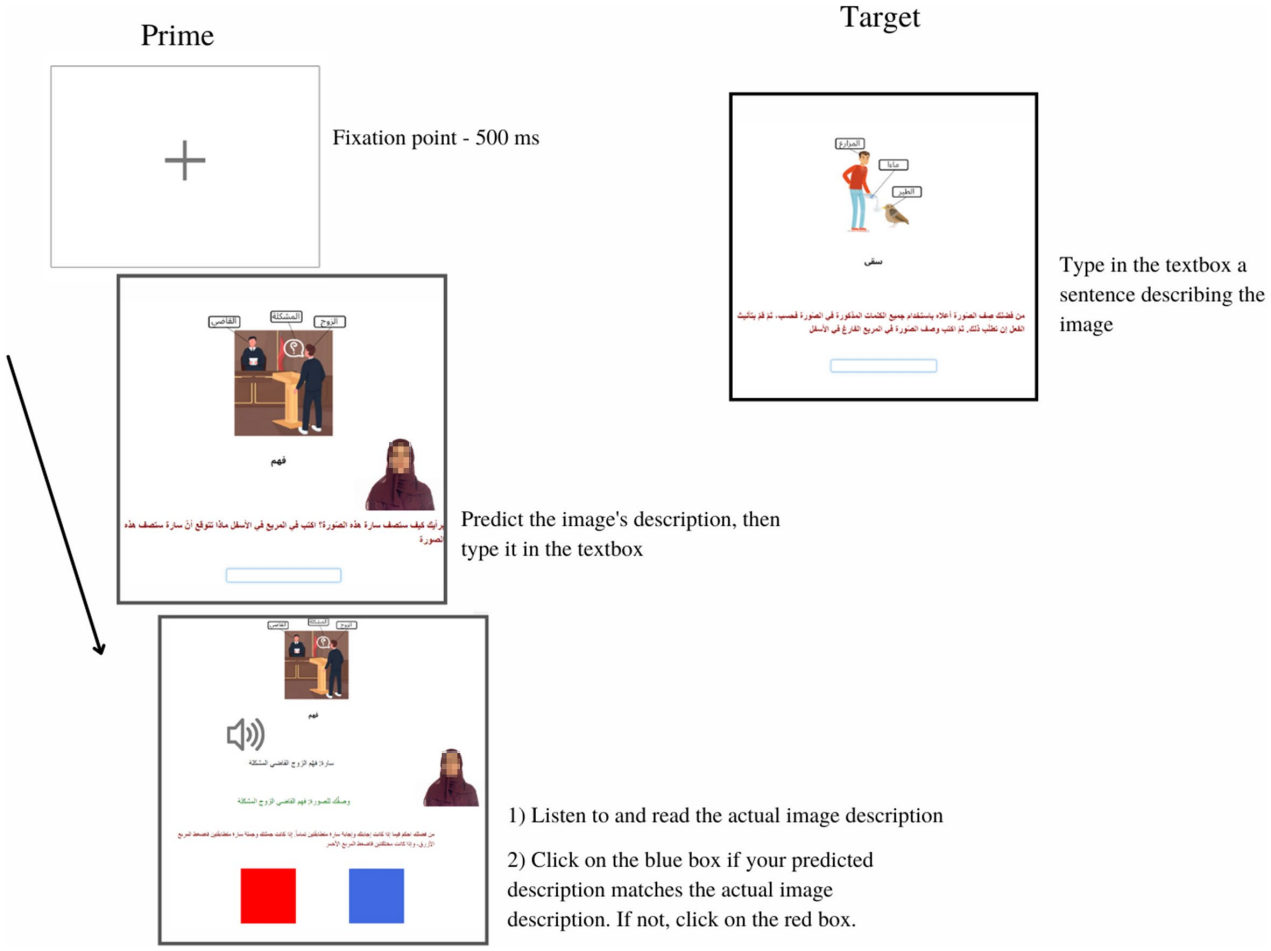
A sample trial in the unconstrained condition in both priming experiments. The priming trial consisted of three screens. In screen one, participants saw a labeled image and were asked to guess how a virtual partner, characterized as a stereotypical Arab woman named “Sarah” would describe it. When participants have finished typing the image description into a textbox, they pressed enter to progress to the next screen. In screen two, participants simultaneously listened to and read the prime sentence. Also, participants saw their image description sentence and Sarah’s actual description and were subsequently asked to “judge whether your sentence and Sarah’s sentence are exactly the same. Click on the blue box if the two sentences are exactly the same. Click on the red box if they are different.” This remained on the screen until participants clicked on either box to progress to the next screen. In screen three, participants saw only a labeled image and were asked to type their own description into a textbox. When finished typing, participants pressed enter to go to the next priming trial. Progress was self-paced.

**Figure 2:**
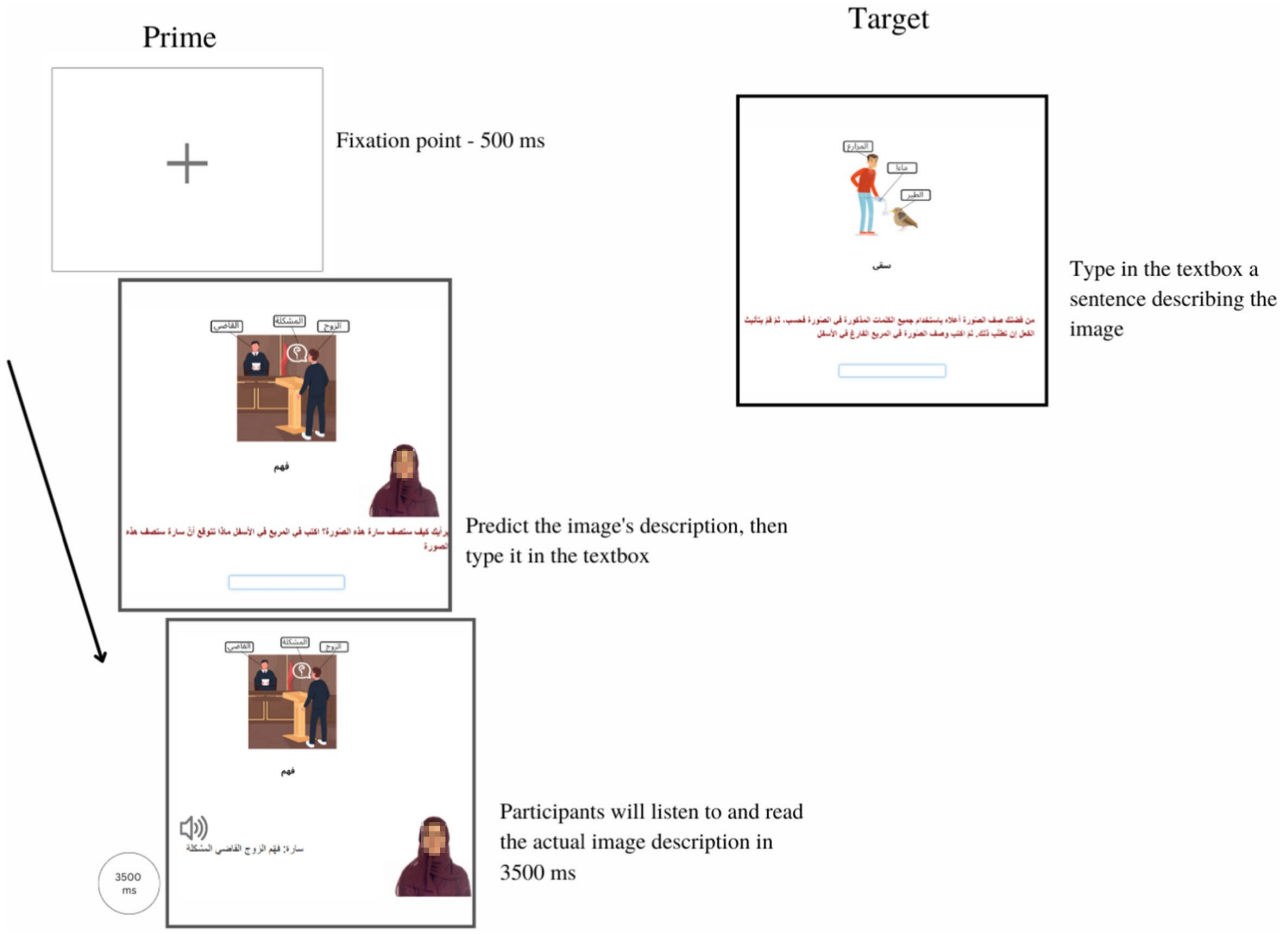
A sample trial in the constrained condition in both priming experiments. There were three screens in the priming trial. Only the second screen was different from the one presented in the unconstrained condition. In the second screen, participants in the constrained condition did not see their image description and were not instructed to compare their guess with the correct answer; instead, they only listened to and read Sarah’s actual answer for 3,500 ms. After the 3,500 ms, participants automatically progressed into the target trial, which replicated the one in the unconstrained condition. Progress was self-paced except for the last prime screen.

**Figure 3:**
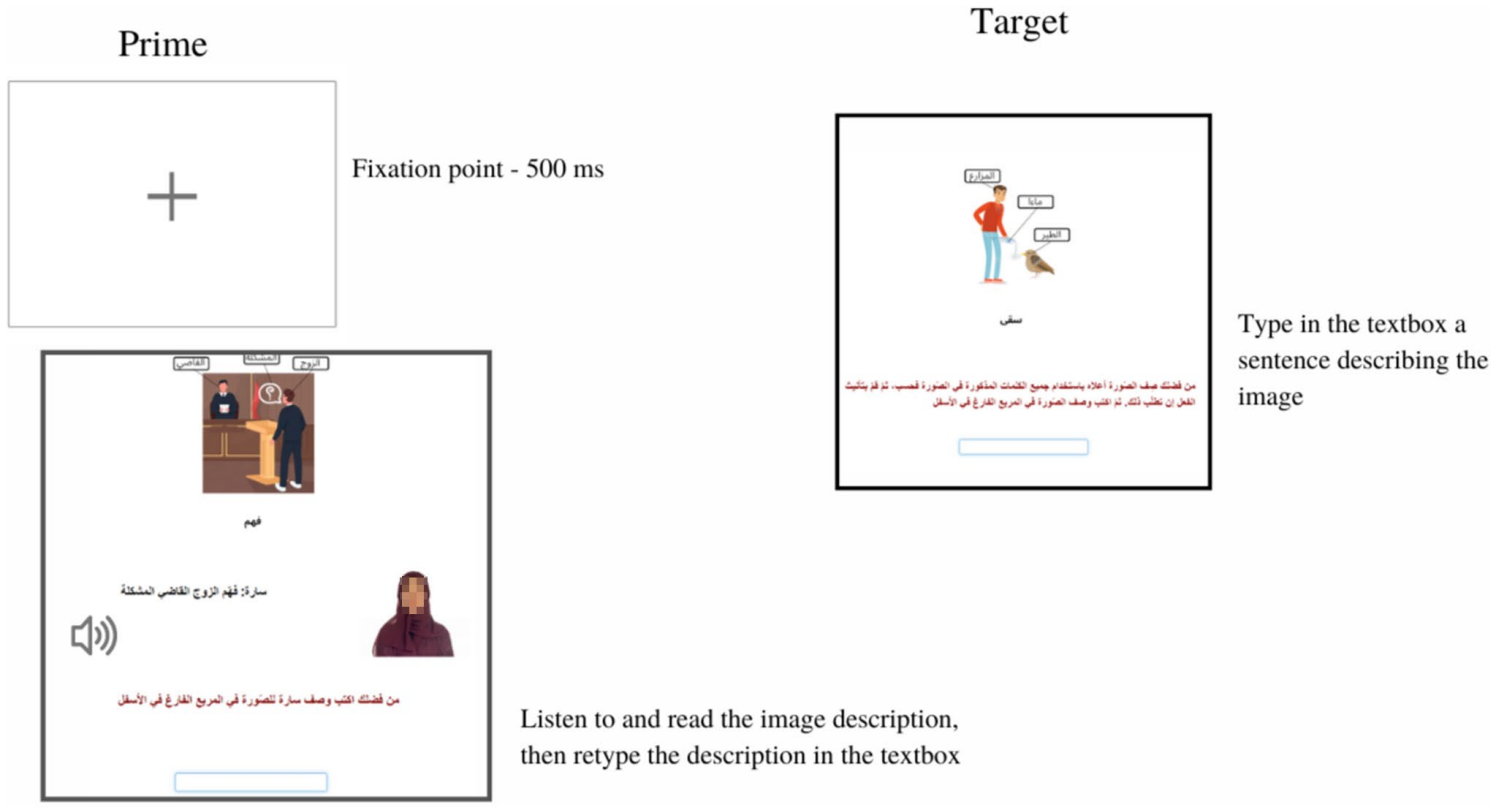
A sample trial in the control condition in both priming experiments. The priming trial consisted only of two screens. In the prime trial, a labeled image was shown, and participants simultaneously listened to and read the prime sentence. While the written prime sentence remained on screen, participants were asked to retype the prime sentence in the textbox and press enter to move to the next screen. In the target trial, participants were shown a labeled image and were asked to type a description into a textbox. Then, participants pressed enter to progress to the remaining trials. Progress was self-paced.

Participants completed the syntactic priming task remotely using the Gorilla.sc platform while being live monitored and assisted by the researcher. Participants first gave their written informed consent and read the task instructions. Then, they completed a two-trial practice session in which they described events using non-target structures to familiarize them with the priming task. After the practice, they were randomly assigned by Gorilla.sc to one of the three priming conditions and completed in the same session three task phases: baseline, priming, and an immediate posttest. Participants completed the three phases in 40–65 min, and they were allowed to take a 30-min to 1-h break between the dative experiment and the TP experiment. Two weeks after the first session, participants remotely completed the delayed posttest, followed by two proficiency tests as well as a background questionnaire. The second part took around 10–20 min to complete. A detailed description of the sequencing of the three conditions is found in Figures 1–3.

## Data analysis

### Scoring

#### Target sentences

Responses on the priming phases for the dative experiment were scored as PO, DO, or “other.” PO sentences included a verb, followed by an agent subject, then an animate recipient and, finally, an inanimate theme that is preceded by the preposition (“إلى”/ila:/to) or (“ل” l:/to). DO sentences included a verb, followed by an agent subject, then an inanimate theme and, finally an animate recipient. The analysis included DO sentences that had a reversed theme/recipient order, in which the recipient preceded the theme, PO sentences that included other less typical prepositions such as (“ب، عن، على”/bi:,ʕәn, ʕәla:/with, for, on), sentences with an added adjective, sentences with a fronted subject, and sentences with switched subject and recipient. Productions with missing arguments, or in a non-dative structure were scored as “other” and excluded from the analyses. For the TP experiment, responses were scored as fronted TP, non-fronted TP, or “other.” Fronted TP sentences started with a TP (preposition, then a noun), followed by a verb, and ended with a subject. Non-fronted TP sentences started with a verb, followed by a subject, and ended with a TP. The analysis included non-fronted TP sentences with added adjectives. Sentences that included a TP with a reversed order such that the noun of the TP preceded the preposition, and incomplete sentences were scored as “other” and excluded from further analysis. Across both experiments, morphological errors, including tense and agreement errors, as well as spelling errors, were ignored (Kaan and Chun, 2017; Grüter et al., 2021; Coumel et al., 2022).

### Statistical modeling

#### The effect of condition on priming and learning

Analyses were conducted using mixed-effect logistic regression models with the package lme4 (Bates et al., 2015) and its Bayesian extension blme (Chung et al., 2013) of R version 4.2.2 since the dependent variable was binary. The TP experiment was analyzed using blme package to overcome complete separation issues (Mansournia et al., 2018) in the TP data: no production of fronted TP in the baseline. The dependent variable in the dative models was the production of DO coded as 0 and PO as 1, while it was the production of non-fronted TP coded as 0 and fronted TP as 1 in the TP models.

The fixed effects for the immediate priming models for both structures included the within-participant variables: prime type (dative models: DO, −0.5, vs. PO, 0.5. TP models: non-fronted TP, −0.5, fronted TP, 0.5), and standardized reading scores (with a mean of 0 and an SD of 0.5). The between-participant variable was condition (control, constrained, unconstrained), which was first sum coded as −0.66, 0.33, 0.33 to compare between the control (−0.66) and two the experimental conditions (0.33), then was coded as 0, −0.50, 0.50 to compare between the constrained (−0.50) and the unconstrained (0.50) conditions. The TP immediate priming included the additional within-participant variable TP placement (TP image placed on the left, −0.5 vs. the right, 0.5). The fixed effects for short-and long-term learning models for both structures included the within-participant variables phase (treatment coded, with the baseline as the reference), and standardized reading scores, as well as the between-participant variable condition (sum contrast coded). Treatment coding was used only for phase following prior practice (Grüter et al., 2021) and to allow the meaningful comparison between the four levels of phase (baseline, priming, immediate posttest, delayed posttest) in one model.

#### The effects of L2 proficiency and structure type

Mixed effect logistic regression models were built to examine the effects of L2 proficiency and structure type on prediction-based learning. Data combining the two experiments was used to model the effects. The dependent variable was the participant’s production of the target structure collapsed across experiments, with PO and fronted TP coded as 1, and DO and non-fronted TP as 0. The fixed effects included the between-participant variable condition (sum contrast coded), as well as the within-participant variables phase (treatment coded, with the baseline as the reference), standardized reading scores for the proficiency model, and experiment (TP, −0.5, dative, 0.5) for the structure type model.

All models started with main effects and their interactions, and random intercepts for participants and items as well as all random slopes justified by the design. Where the maximal model did not converge, random slopes and interactions were removed prior to the main effects. If more than one model converged, they were compared using AIC scores (Cavanaugh and Neath, 2019) and the model with the least scores was reported below.

## Results

### Descriptive statistics

At baseline, participants across conditions produced both DO and PO structures with a preference for DO (Table 1).

**Table 1:** Frequency of target responses for the dative structure by condition, and phase.

Condition	Phase (prime)	Response			
		DO	PO	Other	% of PO
Control (*N* = 51)	Baseline	101	33	19	24.6%
	Priming (DO)	150	29	25	16.2%
	Priming (PO)	144	32	28	18.1%
	Immediate posttest	116	19	18	14%
	Delayed posttest	113	29	11	20.4%
Constrained (*N* = 46)	Baseline	84	31	23	26.9%
	Priming (DO)	104	31	49	22.9%
	Priming (PO)	93	43	48	31.6%
	Immediate posttest	76	33	29	30.2%
	Delayed posttest	97	32	9	24.8%
Unconstrained (*N* = 50)	Baseline	89	41	20	31.5%
	Priming (DO)	202	62	136	23.4%
	Priming (PO)	197	106	97	34.9%
	Immediate posttest	96	38	16	28.3%
	Delayed posttest	121	24	5	16.5%

Percentage of PO = PO/(DO + PO).

Unlike the dative experiment, at baseline, participants across conditions produced either only or mostly non-fronted TP, suggesting a high preference for this structure (Table 2).

**Table 2:** Frequency of target responses for the TP structure by condition, and phase.

Condition	Phase (prime)	Response			
		Non-fronted	Fronted	Other	% of fronted
Control (*N* = 51)	Baseline	150	0	3	0%
	Priming (Non-Fronted)	191	7	6	3.5%
	Priming (Fronted)	157	42	5	21.1%
	Immediate posttest	139	12	2	7.9%
	Delayed posttest	148	3	2	1.9%
Constrained (*N* = 46)	Baseline	132	1	5	0.7%
	Priming (Non-Fronted)	157	17	10	9.7%
	Priming (Fronted)	153	20	11	11.5%
	Immediate posttest	113	22	3	16.2%
	Delayed posttest	134	2	2	1.4%
Unconstrained (*N* = 50)	Baseline	150	0	0	0%
	Priming (Non-Fronted)	349	40	11	10.2%
	Priming (Fronted)	359	35	6	8.8%
	Immediate posttest	128	19	3	12.9%
	Delayed posttest	142	7	1	4.6%

Percentage of fronted TP = fronted TP/(non-fronted TP + fronted TP).

### Between group comparisons

#### Effect of condition on immediate priming

##### PO model

**Figure 4:**
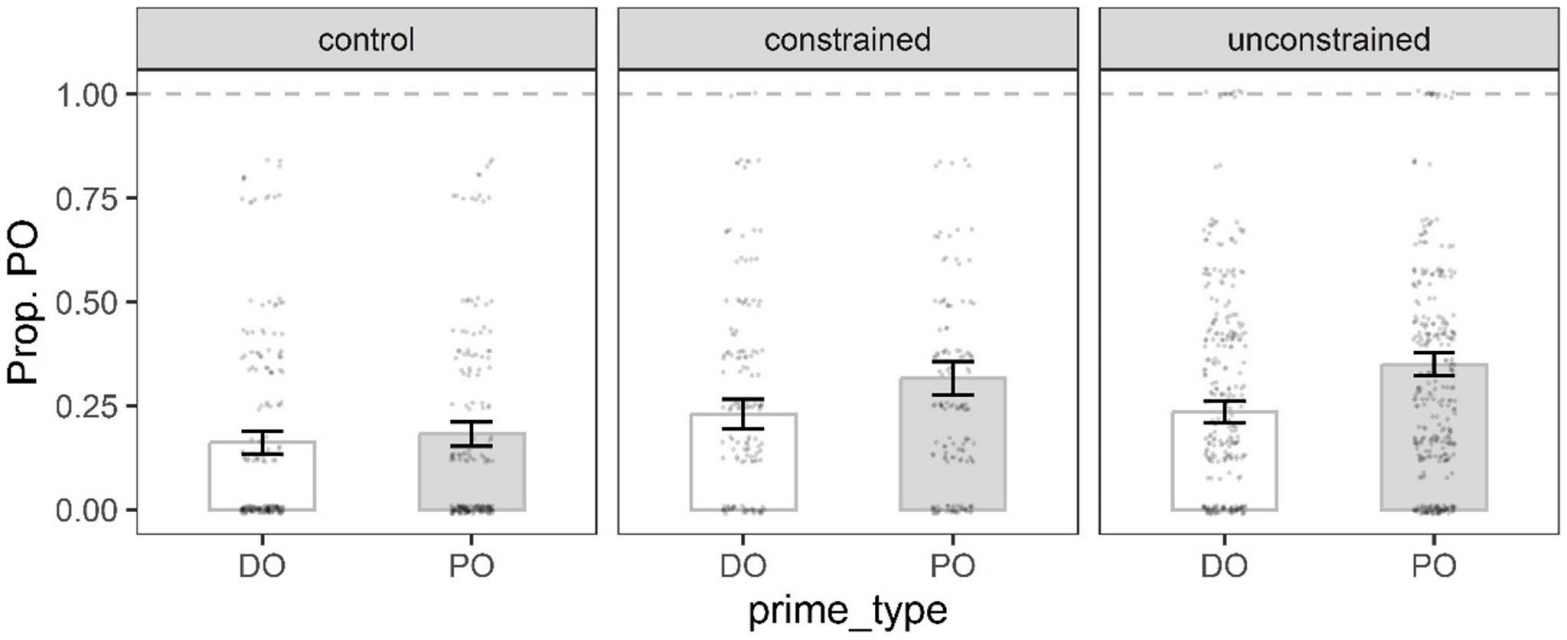
Mean proportion of PO target responses in the priming phase by prime type, and condition. Error bars represent the standard error of the mean, and black dots individual data points.

Table 3 shows the best fit immediate priming model for the PO structure. The significant Condition 1 indicated that participants in the constrained and unconstrained condition produced significantly more PO sentences than DOs in the priming phase compared to the control participants. The significant interaction term Condition 1 x Prime type indicated that participants in both the constrained and unconstrained conditions produced significantly more PO targets following PO primes (31.6, 34.9%, respectively) than following DO primes (22.9, 23.4%, respectively) compared to the controls (Figure 4). The non-significant Condition 2 x Prime type interaction indicated that the production of PO targets after PO primes versus DO primes did not differ between the constrained condition and the unconstrained condition.

**Table 3:** Summary of the best mixed logistic model for PO immediate priming.

Fixed effects	Estimate	95% CI	*SE*	*z*	*p*
Intercept	−1.58	[−2.07, −1.08]	0.25	−6.23	< 0.001
Condition 1	1.01	[0.26, 1.76]	0.38	2.65	0.008
Condition 2	−0.07	[−0.89, 0.76]	0.42	−0.16	0.874
Prime type	0.19	[−0.58, 0.96]	0.39	0.49	0.627
Reading scores	0.12	[−0.20, 0.45]	0.17	0.75	0.454
Condition 1 x Prime type	0.88	[0.06, 1.70]	0.42	2.09	0.036
Condition 2 x Prime type	0.65	[−0.26, 1.56]	0.46	1.39	0.163

Marginal *R*^2^ = 0.047, Conditional *R*^2^ = 0.51.

##### Fronted TP model

**Figure 5:**
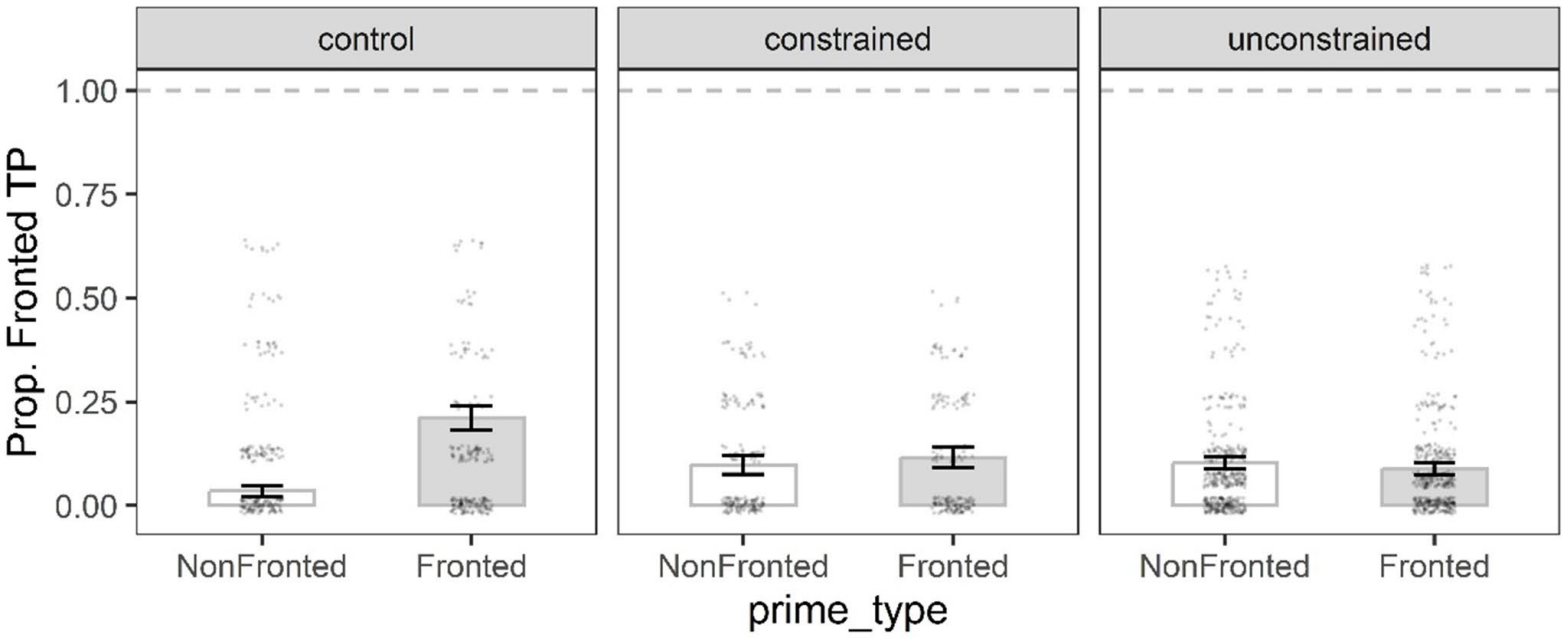
Mean proportion of fronted TP target responses in the priming phase by prime type, and condition. Error bars represent the standard error of the mean, and black dots individual data points.

Table 4 shows the best fit immediate priming model for the fronted TP structure. The significant interaction term Condition 1 x Prime type indicated that the control participants produced significantly more fronted TP targets following fronted TP primes (21.1%) than following non-fronted TP primes (3.5%) compared to the participants in the constrained and unconstrained conditions (Figure 5).

**Table 4:** Summary of the best mixed logistic model for fronted TP immediate priming.

Fixed effects	Estimate	95% CI	*SE*	*z*	*p*
Intercept	−3.02	[−3.70, −2.34]	0.35	−8.73	< 0.001
Condition 1	−0.01	[−0.83, 0.80]	0.42	−0.03	0.974
Condition 2	−0.56	[−1.47, 0.35]	0.47	−1.21	0.227
Prime type	0.31	[−0.69, 1.31]	0.51	0.61	0.542
Reading scores	−0.01	[−0.36, 0.34]	0.18	−0.08	0.939
TP placement (Left vs. right)	−0.19	[−0.66, 0.28]	0.24	−0.79	0.430
Condition 1 x Prime type	−1.91	[−3.01, −0.80]	0.56	−3.39	0.001
Condition 2 x Prime type	0.86	[−0.31, 2.02]	0.60	1.44	0.150

The model was fitted using ‘blme’.

#### Effect of condition on short-and long-term learning

##### PO model

**Figure 6:**
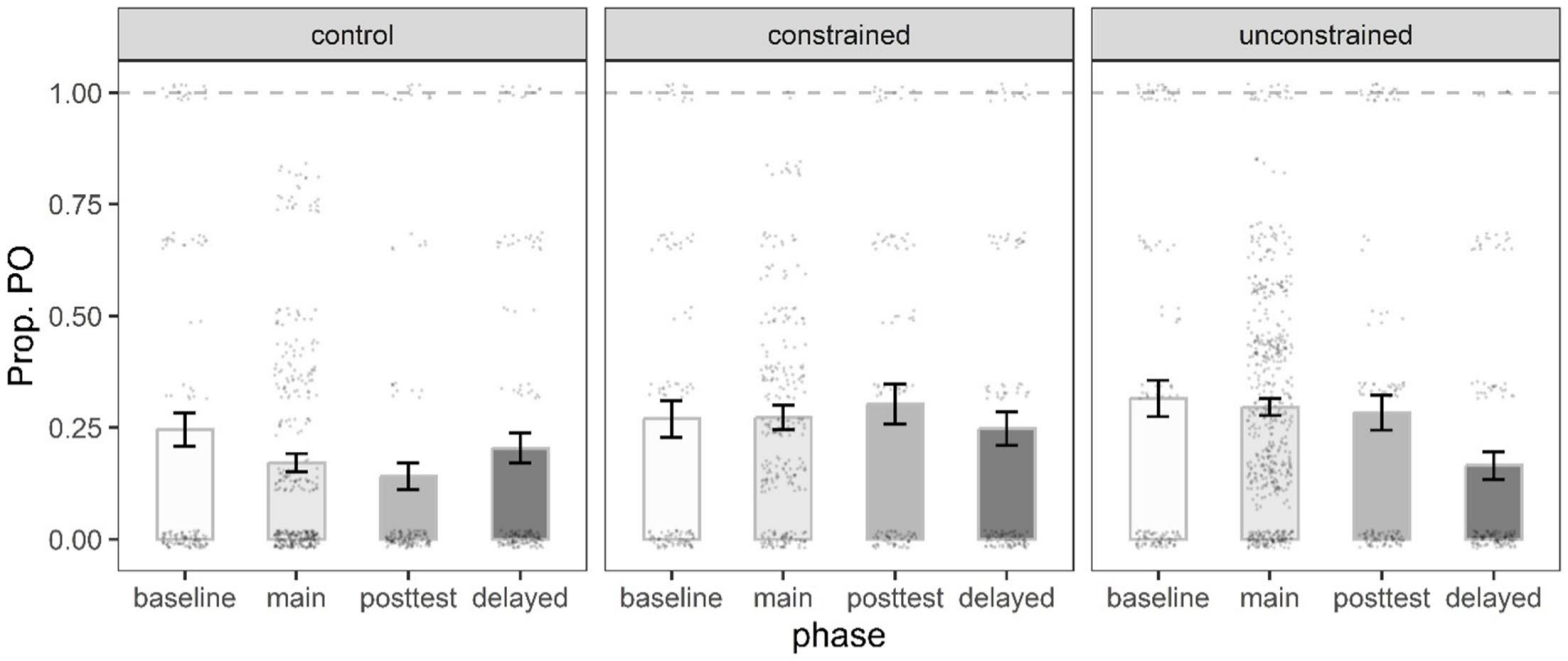
Mean proportion of PO target responses by phase, and condition. Error bars represent the standard error of the mean, and black dots individual data points.

The best-fit PO model had a significant intercept and two significant interactions between phase and condition (Table 5). There was a significant Immediate x Condition 1 interaction suggesting that participants in both the constrained and unconstrained conditions produced more PO sentences in the immediate posttest (30.2, 28.3%, respectively) compared to their counterparts in the control condition (14%). Further, the significant Delayed x Condition 2 interaction indicated that participants in the unconstrained condition produced far less PO sentences (16.5%) in the delayed posttest compared to those in the constrained condition (24.8%) (Figure 6).

**Table 5:** Summary of the best mixed logistic model for PO priming across phases and conditions.

Fixed effects	Estimate	95% CI	*SE*	*z*	*p*
Intercept	−1.36	[−2.10, −0.63]	0.38	−3.62	< 0.001
Priming phase	−0.21	[−0.97, 0.55]	0.39	−0.53	0.594
Immediate posttest	−0.31	[−1.30, 0.67]	0.50	−0.63	0.532
Delayed posttest	−0.55	[−1.53, 0.44]	0.50	−1.09	0.276
Condition 1	0.54	[−0.24, 1.32]	0.40	1.36	0.172
Condition 2	0.40	[−0.47, 1.27]	0.44	0.90	0.368
Priming x Condition 1	0.60	[−0.09, 1.28]	0.35	1.69	0.090
Immediate x Condition 1	1.06	[0.19, 1.93]	0.44	2.39	0.017
Delayed x Condition 1	−0.29	[−1.11, 0.52]	0.42	−0.70	0.482
Priming x Condition 2	−0.32	[−1.06, 0.42]	0.38	−0.85	0.397
Immediate x Condition 2	−0.48	[−1.36, 0.39]	0.45	−1.08	0.281
Delayed x Condition 2	−1.09	[−2.00, −0.19]	0.46	−2.37	0.018

Marginal R^2^ = 0.048, Conditional R^2^ = 0.45.

##### Fronted TP model

**Figure 7:**
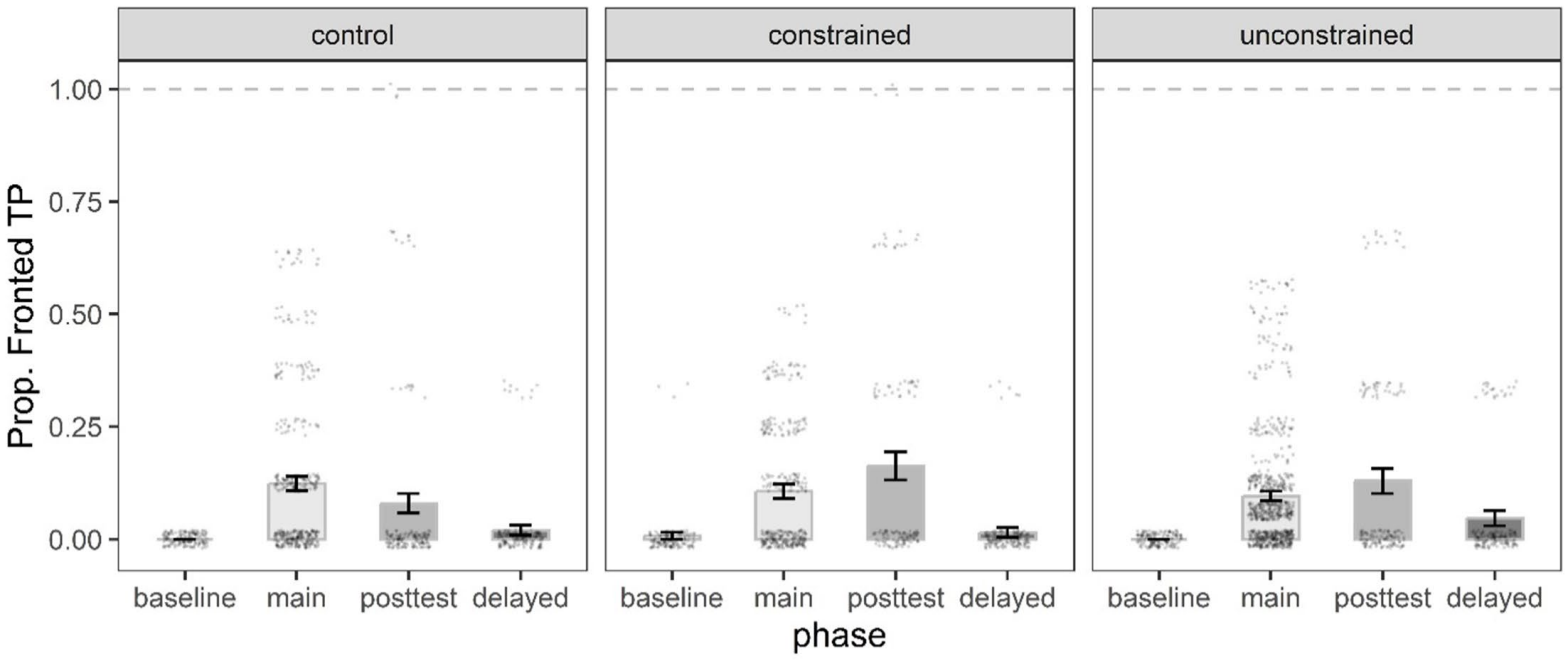
Mean proportion of Fronted TP target responses by phase, and condition. Error bars represent the standard error of the mean, and black dots individual data points.

Meanwhile, the best-fit fronted TP model had a significant intercept and two significant simple effects (Table 6). The significant priming and immediate posttest effects indicated that on average participants across conditions (control, constrained, unconstrained) produced significantly more fronted TPs in both the priming phase (24.6, 21.2, 19%, respectively) and the immediate posttest (7.9, 16.2, 12.9%, respectively) compared to the baseline (0, 0.7, 0%, respectively) (Figure 7). There were no significant fronted TP responses in the delayed phase relative to the baseline across conditions. Importantly, there were no significant differences between conditions in short-and long-term learning for the fronted TP structure (*p* > 0.05).

**Table 6:** Summary of the best mixed logistic model for fronted TP priming across phases and conditions.

Fixed effects	Estimate	95% CI	*SE*	*z*	*p*
Intercept	−5.85	[−7.25, −4.44]	0.72	−8.14	< 0.001
Priming phase	2.88	[1.44, 4.32]	0.74	3.92	< 0.001
Immediate posttest	3.20	[1.59, 4.81]	0.82	3.89	< 0.001
Delayed posttest	1.26	[−0.45, 2.97]	0.87	1.44	0.149
Condition 1	0.55	[−1.58, 2.68]	1.09	0.50	0.614
Condition 2	−0.66	[−2.82, 1.50]	1.10	−0.60	0.551
Priming x Condition 1	−0.94	[−3.06, 1.18]	1.08	−0.87	0.383
Immediate x Condition 1	0.33	[−1.85, 2.51]	1.11	0.30	0.766
Delayed x Condition 1	−0.08	[−2.49, 2.33]	1.23	−0.06	0.949
Priming x Condition 2	0.09	[−2.06, 2.25]	1.10	0.08	0.934
Immediate x Condition 2	0.30	[−1.90, 2.49]	1.12	0.27	0.790
Delayed x Condition 2	1.73	[−0.76, 4.22]	1.27	1.36	0.173

The model was fitted using ‘blme’.

##### Effect of L2 proficiency

A mixed logistic regression model was fitted to examine the effect of L2 proficiency (reading test scores) on immediate priming and short-and long-term syntactic learning across structures. No significant effect emerged in the proficiency model.

### Within individual comparisons

#### Effect of structure type

**Figure 8:**
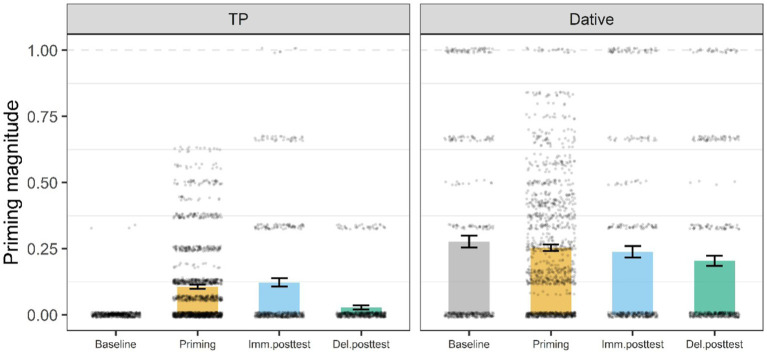
Mean proportion of target responses (Fronted TP, PO) by experiment and phase. Error bars represent the standard error of the mean, and black dots individual data points. Imm.posttest, immediate posttest; Del.posttest, delayed posttest.

A mixed logistic model was fitted to examine the effect of structure type (fronted TP versus PO) on immediate priming, short-and long-term learning across conditions and experiments. There was a significant effect for Experiment (*β* = −3.22, 95% CI [−4.28, −2.16], *SE* = 0.54, *z* = −5.93, *p* < 0.001), which showed that participants across conditions and experiments produced more PO sentences in the baseline than fronted TPs. However, the significant two interactions between Experiment and phase indicated that participants across conditions and experiments produced more fronted TP responses than PO responses in the priming phase (*β* = −12.11, 95% CI [−23.81, −0.42], *SE* = 5.97, *z* = −2.03, *p* < 0.042) as well as the immediate posttest phase (*β* = −12.34, 95% CI [−24.06, −0.62], *SE* = 5.98, *z* = −2.06, *p* < 0.039) compared to the baseline. Crucially, the experimental conditions did not significantly differ from one another nor from the control across experiments (Figure 8).

## Discussion

The implicit learning account hypothesizes that prediction derives L1 and L2 language acquisition through the computation of prediction errors (Chang et al., 2006). This hypothesis was tested in the present study by disentangling the effect of guessing (the constrained condition) and the effect of prediction error computation (the unconstrained condition) on prediction-based learning. Further, the effects of syntactic complexity and L2 proficiency were investigated. A summary of significant effects is presented in Table 7. The results of this study show that the experimental conditions performed similarly across the structures, unlike the control. This pattern of results is inconsistent with the implicit learning account as well as previous research (Grüter et al., 2021), which expected a difference between the experimental conditions. These findings are elaborated on below as well as their implications for related theories.

**Table 7:** Summary of key significant results.

Structure type	Type of learning	Constrained	Unconstrained	Control
PO	Immediate priming	YES	YES	
	Short-term			
	Long-term			
Fronted TP	Immediate priming			YES
	Short-term	YES	YES	
	Long-term			

### Differences between the conditions

The implicit learning account predicts that participants who engage in error computation (unconstrained condition) would show improved learning, while there was no similar prediction for making a guess only (constrained condition). As such, the unconstrained condition should show larger priming and learning. However, both experimental conditions, compared to the control, showed a similar pattern of effects: enhanced immediate priming for the same structure (PO) as well as significant short-term learning for one structure (fronted TP). Since the constrained condition involved (a) making a guess only while restricting (b) the consequent computation of prediction error, yet performed similarly to the unconstrained condition which required both (a) and (b), it is reasonable to posit a positive and separate effect of guessing on priming and learning. To my knowledge, the current study is the first to find a guessing benefit for syntactic priming and learning over and above the computation of prediction error. This guessing advantage could elucidate some of the contributions of prediction in L2 processing and learning, and such information could help us in creating more optimal L2 learning tasks.

The current findings suggest that an L2 speaker who is instructed to guess the structure of the next utterance is more likely to be more attentive while processing that utterance regardless of an explicit instruction to engage in error computation. Such memory enhancement is explained in the neuroscience literature in terms of state curiosity (Gruber and Ranganath, 2019). There is some evidence from this literature that individuals demonstrate better memory and retention of answers to questions that elicited high levels of curiosity (van Lieshout et al., 2018). In the present study, participants in the experimental conditions could have experienced larger immediate priming and learning due to being in a heightened curiosity state. Making a guess may have increased the speakers’ desire to know the correct answer and this in turn have led to deeper encoding of that answer (Potts and Shanks, 2014; Potts et al., 2019). This increased attention to the correct answer is believed to be motivated by the intrinsic reward value of knowing the actual answer (Gruber and Ranganath, 2019; Gambi, 2021). Learning and priming in this case becomes reward-driven such that the participants wanted to find evidence to help confirm their predictions, and this search for a confirmation (reward) fostered a change in syntactic preferences over time (i.e., after trials/a session/weeks). Previous research has also observed that people placed in high curiosity contexts show enhanced learning for newly acquired L1 vocabulary items compared to those being placed in low curiosity contexts (Potts et al., 2019). The current study has extended this observation to prediction in L2 learning research and showed that curiosity could affect L2 speakers’ sensitivity to confirmed/disconfirmed predictions, resulting in short-lived changes in existing syntactic representations.

The reported guessing benefit on syntactic priming and learning did not persist over time, as found in the 2-week delayed test. The experimental conditions did not experience long-term learning for either structure type. The limited long-term learning across the experimental conditions may be attributed to the use of an insufficient number of items across priming (*n* = 16), immediate posttest (*n* = 3), and delayed posttest (*n* = 3). A previous L2 syntactic priming study which reported sustained long-term learning has included more items in the priming phase (*n* = 48), immediate posttest (*n* = 18), as well as the in the one-week delayed posttest (*n* = 12) (Coumel et al., 2022). Two further reasons could explain the unconstrained condition findings. One possibility is that the prediction error feedback in the unconstrained condition might have introduced a cognitive load for the pre/upper intermediate L2 speakers, which prevented the durable shift in structural preferences (Zhang et al., 2020). But if there was a cognitive load, then there should be no evidence for short-term learning. Another possibility is the prediction error feedback in the unconstrained condition could have stalled memory retrieval due to competition in memory from the wrong predictions (Wang et al., 2022). While some of these explanations might apply, it is difficult to interpret null results. The reason for not observing long-term learning across the experimental conditions remains unknown.

Findings from the experimental conditions have implications for the implicit learning account. As discussed earlier, a common assumption has been that tacit prediction derives learning through the computation of prediction errors so that erroneous predictions lead the speaker to make changes to the linguistic system (Chang et al., 2006). On this view, engagement in error computation should lead to better priming and learning than when such engagement is constrained. In the current study, durable priming and learning occurred even in the constrained condition in which L2 speakers were required to predict but were not offered sufficient time to compare their predictions with and the actual input (i.e., a time limit of 3,500 ms to view the actual sentence). Considering the observed impairments in sentence comprehension under the 3,500 ms condition in the picture-sentence matching task (see Method), it was unlikely that participants in the constrained condition of the priming task were able to fully engage in prediction error. In this case, generating a prediction only seems to have benefited priming and learning, possibly via increased curiosity. Thus, this guessing benefit may suggest some role for curiosity in L2 priming and learning. The effect of curiosity state on syntactic learning and consolidation, nevertheless, was not considered in the implicit learning account. A more comprehensive picture of prediction in L2 learning could be obtained by considering the effect of guessing and the subsequent increased curiosity on sentence processing and learning.

Another striking result was the lack of short and long-term learning in the control condition across structures as assessed in the immediate posttest and 2-week delayed test, respectively. This is incompatible with previous L2 studies that used the same repetition priming task and found increased production of the target structure in immediate and delayed tests (1 day to 1 week) relative to the baseline (Jackson and Hopp, 2020; Grüter et al., 2021; Coumel et al., 2022). Two reasons could explain this divergence. Some of these earlier works have reused the same verbs across phases (Coumel et al., 2022), and lexical repetition is known to increase the magnitude of priming and subsequent learning (Mahowald et al., 2016). The present study, on the other hand, used unique verbs for each priming phase, with the exception of only one verb that was repeated between the immediate and delayed posttest. Another potential reason is that the participants in the present study may not have been suitably proficient in their L2 to show an abstract syntactic priming effect compared to those in previous works (Jackson and Hopp, 2020; Grüter et al., 2021). This is especially the case since less proficient L2 speakers may be less susceptible to abstract syntactic priming in which the prime and target sentences do not share lexical heads (Bernolet et al., 2013; Hartsuiker and Bernolet, 2017).

### Structure type

Results showed that all conditions demonstrated differential priming and learning effects that varied according to the type of prime structure. This variation was observed within and across groups. This is one of the few studies, to my knowledge, that compared prediction-based learning for two different structures within L2 speakers. Existing works have instead investigated the effect of prediction on learning a single syntactic structure (Grüter et al., 2021) or two structures that varied in ambiguity (Kaan et al., 2019; Chun et al., 2021). As such, the present study makes important contributions to the literature on L2 prediction-based learning across different syntactic structures.

There were clear group-level differences modulated by the prime structure type. For example, the experimental conditions manifested immediate priming for PO exclusively, while the controls experienced the opposite result: immediate priming only for fronted TP. The priming of two different structures to the same individuals allows us to conclude that the observed variations are likely due to condition. The implicit learning account is not compatible with the observed within and across individual variation (Chang et al., 2006). The utility approach, on the other hand, could accommodate these findings (Kuperberg and Jaeger, 2016). According to this approach, the distinctive goals and constraints of each condition could have contributed to such within and across group differences.

This pattern of results for the effect of structure type could be attributed to the use of distinct priming mechanisms across conditions. Participants across conditions seem to have utilized different types of processes to comprehend the prime sentences. Recall that in the baseline, participants across conditions uniformly produced a considerable number of PO sentences (range = 31–41) but rarely produced fronted TPs (range = 0–1), suggesting that PO was more frequently used than fronted TPs and more familiar for the participants (see Tables 1, 2). A more frequently used structure has higher base-level activation (i.e., sufficient productive knowledge) compared to one that is less frequently produced (Reitter et al., 2011). Crucially, structures with intermediate base-level seem to show larger immediate priming under guessing conditions. Two findings from the experimental conditions could support this idea. One is the significant immediate priming of PO, which seems to have occurred because participants demonstrated some level of productive PO knowledge in the baseline. The second is the observation that fronted TP was only successfully primed in the immediate posttest, after sufficient exposure and production of fronted TP in the priming phase, which increased its activation levels in explicit memory. Overall, this suggests that structures with existing base-level activation are more likely to trigger priming and learning in experimental conditions.

The unique priming of the more frequent structure (PO) in the experimental conditions does not fit with the implicit learning account. Under this account, syntactic priming is assumed to be based on the non-declarative, implicit memory system that is prone to show increased priming for less frequently encountered structures. However, the experimental condition enhanced priming for the more frequent structure. Assuming a relationship between the use of implicit memory and the priming of less frequent structures, the findings from the experimental condition could indicate a larger role for explicit memory. Support for the predominance of declarative, explicit memory in guessing situations comes from neuroscientific research showing that a heightened curiosity state, as induced when participants are asked to guess the answer of trivia questions, leads to a search for a relevant answer in explicit memory (Gruber and Ranganath, 2019). Thus, the instruction to guess in the current study may have encouraged L2 speakers to activate their existing syntactic representations and look for the expected structure in their memory.

Meanwhile, the control participants manifested priming for the less-frequent structure (fronted TP), which had less established baseline knowledge. This finding is compatible with the well-established inverse frequency effect in the L2 syntactic priming literature, in which less frequent syntactic structures are expected to show larger priming (Kaan and Chun, 2017; Montero-Melis and Jaeger, 2020; Muylle et al., 2021). This study extended the inverse-frequency effect to a new L2 group: L2 Arabic speakers, contributing by this to the L2 syntactic priming research. Unlike the experimental conditions, priming in the control is largely consistent with the implicit learning account. Control participants showed priming patterns that suggest a role for an implicit learning mechanism that is more susceptible to the priming of less frequent syntactic representations.

These findings have two significant implications for syntactic priming and prediction-based learning research. First, the mechanism underlying priming and learning in a guessing situation could be attributed to the primary use of explicit memory. Forming a prediction regarding the syntax of the next utterance might lead L2 speakers to consult their existing syntactic knowledge using the explicit memory system, resulting in short learning. The present study is one of the few works that demonstrated the persistence of priming effects via an explicit learning mechanism. The prominent implicit learning account does not fit with the current findings. This account suggests that durable priming mainly arises from the use of the implicit learning mechanism. Similarly, other syntactic priming accounts propose a role for explicit memory in syntactic priming effects, but maintain that priming effects mediated by an explicit memory are short-lived (Reitter et al., 2011; Segaert et al., 2016; Heyselaar et al., 2021). Thus, a more comprehensive syntactic priming account is needed to accommodate the present data.

Second, a guessing benefit in a syntactic priming task may extend to L2 speakers who demonstrate some productive knowledge of the prime structure since making a guess seems to require a search in explicit memory. Based on current suggestive evidence (numerical baseline data; Tables 1, 2), such benefits seem to extend to an L2 speaker who has basic syntactic knowledge of the prime structure but might not occur for that speaker when she has a diminished syntactic representation for the prime. To illustrate, participants in the experimental conditions generated a considerable number of PO sentences in the baseline (range = 31–41) and showed immediate PO priming. On the other hand, the experimental conditions produced few to no fronted TPs in the baseline (range = 0–1) and did not experience immediate priming for TPs. Combined, such findings imply that some form of productive knowledge for the prime is a prerequisite for the occurrence of a guessing effect or prediction-based learning. These findings are consistent with the utility approach to linguistic prediction (Kuperberg and Jaeger, 2016) which suggests that language speakers efficiently engage with prediction only when they have the required syntactic knowledge.

Current results could also shed light on the role of structure complexity in prediction-based learning via syntactic priming. As was mentioned above, the complexity of the Arabic dative structure, which contains more constituents than the fronted TP structure (as depicted in Supplementary Figure S1), has been suggested to pose a challenge for L2 Arabic speakers (Al-Jadani, 2016). Surprisingly, despite the presumed higher complexity of the PO dative, L2 participants in the experimental conditions exhibited priming for this structure but not for the less complex fronted TP construction during the priming phase. One way to explain this result is to consider structure familiarity or frequency. As demonstrated in the baseline, the L2 speakers were familiar with the PO dative in that they produced a considerable number of PO sentences (range = 31–41; 24–31%) but did not show a similar degree of familiarity with fronted TPs (range = 0–1; 0–0.7%). Thus, structure frequency or familiarity seems to play a key role in triggering prediction-based learning, but there is limited evidence for a similar effect of structure complexity. Yet, this limited evidence cannot rule out the potential effects of structure complexity in prediction-based learning and future research needs to investigate this topic more systematically.

### L2 proficiency

This study found no effect for Arabic L2 proficiency within or across group. Participants benefited from making a guess regardless of their proficiency level as measured in a MCQ Arabic test. This contradicts the predictions of the implicit learning account and the utility approach, with both positing a role for proficiency. Two reasons could explain the null proficiency effect. The used proficiency measure may have lacked the appropriate level of reliability (Cronbach’s alpha = 64, omega = 72), impacting its validity to capture variance in L2 proficiency. Another possibility is that the recruited participants might have had limited variability in their Arabic language skills, making it difficult to find an observable proficiency effect. While these factors could have contributed to a non-significant proficiency effect, this null effect remains difficult to explain.

### Practical implications

The results of this study have clear implications for L2 instruction. The used syntactic priming task includes a guessing game activity that could directly be applied in L2 grammar lessons and materials. This guessing activity may promote increased production, appropriate use, and retention of the target syntactic structure. The guessing task augments syntactic learning and appears to be effective when L2 speakers have some knowledge of the target structure. Thus, the task could be successfully implemented following the introduction of the target structure(s) rather than prior to establishing a basic understanding of that structure. Incorporating a syntactic guessing game in L2 lessons and textbooks has the potential of gamifying L2 syntactic learning and in turn boosting engagement, resulting in improved learning (Castillo-Cuesta, 2020; Sadeghi et al., 2022; Zhang and Hasim, 2022).

## Limitations and future directions

The current work found that generating a guess may trigger short-term L2 learning but does not seem to lead to durable L2 learning. Future works may use a larger number of items and/or recruit more proficient L2 speakers to better assess the guessing benefit on consolidating L2 syntactic information. Similarly, no significant role for L2 proficiency was observed in the present study. One topic for future research should be to explore whether the lack of a significant proficiency effect is due to limitations in measurement and/or sampling in the current study or because global L2 proficiency does not significantly influence prediction-based learning. Current findings numerically suggested a role for L2 syntactic knowledge in prediction-based learning. Subsequent research may directly examine this effect by devising a suitable experimental manipulation. The effect of L1 on prediction-based learning was not a main focus in the present work due to the limited number of Arabic L2 speakers who share the same L1 at the recruitment site. Investigating the effect of L1 by looking at a specific language pair, such as L1 Mandarin and L2 Arabic, may further elucidate the factors that mediate prediction-based learning. Further, the syntactic priming task was conducted online with live remote monitoring by the researcher due to logistic issues. A problem with an online task is the possibility of reduced participant attention. Future studies should ensure task attention by assessing participants’ accuracy in forced-choice comprehension questions during the syntactic priming task (Bernolet et al., 2013; Jackson and Hopp, 2020; Coumel et al., 2022). A further drawback is that the order of the two priming experiments was not counterbalanced (e.g., Coumel, 2021). However, the use of three conditions here may have guarded against this issue. For example, the controls experienced immediate priming only in the later TP experiment, whereas the experimental conditions showed immediate priming in the earlier dative experiment but not in the subsequent TP experiment. These findings may highlight the relative importance of structure type in inducing an immediate priming effect, and the limited role of experiment order in triggering such an effect. Nevertheless, ideally studies should counterbalance experiments to minimize the practice effect. Thus, future studies should consider counterbalancing the order of experiments.

Finally, the present work examined whether explicit prediction could improve learning, as implicit prediction is thought to do. Consequently, it remains unknown whether and how conscious and unconscious computation of prediction errors interact in a language learning task. The next question to explore would be how explicit and implicit processes are related when learning a syntactic structure to determine if explicit processes are facilitative or inhibitive to implicit learning.

## Conclusion

Results from the current study suggest that Arabic L2 speakers at different proficiency levels showed enhanced priming and short-term learning for two syntactic structures (PO, fronted TP) when (a) instructed to guess only (constrained condition) as well as when (b) instructed to guess and compute the prediction error (unconstrained condition). These results hint at a guessing benefit for priming and short-term learning that is seemingly distinguished from the benefit of resolving prediction error. Current evidence further points out a larger role for explicit memory compared to implicit memory in L2 prediction-based learning. This is one of the few studies that found a positive and separate role for guessing on L2 syntactic priming and learning and reported the perseverance of syntactic priming via an explicit learning mechanism. The influential implicit learning account does not accommodate these two findings. The present data could be better explained by a model that accounts for the speaker-related factor curiosity in L2 syntactic priming and learning.

## Data availability statement

The datasets presented in this study can be found in online repositories. The names of the repository/repositories and accession number(s) can be found in the article/Supplementary material.

## Ethics statement

The studies involving human participants were reviewed and approved by the Humanities and Social Sciences Research Ethics Committee at King Saud University. The patients/participants provided their written informed consent to participate in this study. Written informed consent was obtained from the individual(s) for the publication of any potentially identifiable images or data included in this article.

## Author contributions

The author confirms being the sole contributor of this work and has approved it for publication.

## Funding

This research was funded by King Saud University.

## Conflict of interest

The author declares that the research was conducted in the absence of any commercial or financial relationships that could be construed as a potential conflict of interest.

## Publisher’s note

All claims expressed in this article are solely those of the authors and do not necessarily represent those of their affiliated organizations, or those of the publisher, the editors and the reviewers. Any product that may be evaluated in this article, or claim that may be made by its manufacturer, is not guaranteed or endorsed by the publisher.

## References

[ref1] Al Masaeed K. Taguchi N. Tamimi M. (2020). Proficiency effects on L2 Arabic refusals: appropriateness, linguistic strategies and multidialectal practices. Appl. Pragmat. 2, 26–53. doi: 10.1075/ap.19007.mas

[ref2] Alhawary M. T. (2019). Arabic second language learning and effects of input, transfer, and typology. Washington, DC: Georgetown University Press.

[ref3] Al-Jadani A. S. R. (2016). Second language Acquisition of the Dative Alternation in English and Arabic: a bidirectional study (doctoral dissertation) Available at: https://etheses.whiterose.ac.uk/13730/ (Accessed January 20, 2020)

[ref4] Anwyl-Irvine A. L. Massonnié J. Flitton A. Kirkham N. Evershed J. K. (2020). Gorilla in our midst: an online behavioral experiment builder. Behav. Res. Methods 52, 388–407. doi: 10.3758/s13428-019-01237-x, PMID: 31016684 PMC7005094

[ref5] Bates D. Maechler M. Bolker B. Walker S. (2015). Fitting-linear mixed-effects models using LME 4. J. Stat. Softw. 67, 1–48. doi: 10.18637/jss.v067.i01

[ref6] Bernolet S. Hartsuiker R. Pickering M. J. (2013). From language-specific to shared syntactic representations: the influence of second language proficiency on syntactic sharing in bilinguals. Cognition 127, 287–306. doi: 10.1016/j.cognition.2013.02.005, PMID: 23548434

[ref7] Bock J. K. (1986). Syntactic persistence in language production. Cogn. Psychol. 18, 355–387. doi: 10.1016/0010-0285(86)90004-6, PMID: 36856897

[ref8] Bovolenta G. Marsden E. (2021a). Expectation violation enhances the development of new abstract syntactic representations: evidence from an artificial language learning study. Lang. Dev. Res. 1, 193–243. doi: 10.34842/c7t4-pz50

[ref9] Bovolenta G. Marsden E. (2021b). Prediction and error-based learning in L2 processing and acquisition: a conceptual review. Stud. Second. Lang. Acquis. 44, 1–26. doi: 10.1017/S0272263121000723

[ref201] Branigan H. P. Gibb C. L. (2018). Structural priming. In. Research methods in psycholinguistics and the neurobiology of language: A practical guide eds. A. M. B. de Groot and P. Hagoort (pp. 130–150). Wiley Blackwell.

[ref10] Castillo-Cuesta L. (2020). Using digital games for enhancing EFL grammar and vocabulary in higher education. Int. J. Emerg. Technol. Learn. 15, 116–129. doi: 10.3991/ijet.v15i20.16159

[ref11] Cavanaugh J. E. Neath A. A. (2019). The Akaike information criterion: background, derivation, properties, application, interpretation, and refinements. WIREs Comput. Statis. 11, 1–11. doi: 10.1002/wics.1460

[ref12] Chang F. Dell G. S. Bock K. (2006). Becoming syntactic. Psychol. Rev. 113, 234–272. doi: 10.1037/0033-295X.113.2.234, PMID: 16637761

[ref13] Chang F. Fitz H. (2014). “Computational models of sentence production: a dual-path approach” in The Oxford handbook of language production. eds. Goldrick M. Ferreira V. S. Miozzo M. (Oxford: Oxford University Press), 1–30.

[ref14] Chen X. Wang S. Hartsuiker R. (2022). Error-based structure prediction in language comprehension: evidence from verb bias effects in a visual-world structural priming paradigm for mandarin Chinese. J. Exp. Psychol. Learn. Mem. Cogn. 48, 60–71. doi: 10.1037/xlm0001048, PMID: 34410806

[ref15] Chun E. Chen S. Liu S. Chan A. (2021). “Influence of syntactic complexity on second language prediction” in Prediction in second language processing and learning. eds. Kaan E. Grüter T. (Amsterdam: John Benjamins), 70–89.

[ref16] Chung Y. Rabe-Hesketh S. Dorie V. Gelman A. Liu J. (2013). A nondegenerate penalized likelihood estimator for variance parameters in multilevel models. Psychometrika 78, 685–709. doi: 10.1007/s11336-013-9328-2, PMID: 24092484

[ref17] Coumel M. (2021). An exploration of second language learning via syntactic priming (Doctoral dissertation, University of Warwick).

[ref18] Coumel M. Ushioda E. Messenger K. (2022). Second language learning via syntactic priming: investigating the role of modality, attention, and motivation. Lang. Learn. 73, 231–265. doi: 10.1111/lang.12522

[ref19] Dell G. S. Chang F. (2014). The P-chain: relating sentence production and its disorders to comprehension and acquisition. Philosoph. Trans. Royal Soc. B 369:394. doi: 10.1098/rstb.2012.0394, PMID: 24324238 PMC3866424

[ref20] Dempsey J. Liu Q. Christianson K. (2020). Convergent probabilistic cues do not trigger syntactic adaptation: evidence from self-paced reading. J. Exp. Psychol. Learn. Mem. Cogn. 46:881. doi: 10.1037/xlm0000881, PMID: 32551745

[ref21] Fazekas J. Jessop A. Pine J. Rowland C. (2020). Do children learn from their prediction mistakes? A registered report evaluating error-based theories of language acquisition. Royal Soc. Open Sci. 7:180877. doi: 10.1098/rsos.180877, PMID: 33391776 PMC7735343

[ref22] Fine A. B. Jaeger T. F. (2013). Evidence for implicit learning in syntactic comprehension. Cogn. Sci. 37, 578–591. doi: 10.1111/cogs.12022, PMID: 23363004

[ref23] Fine A. B. Jaeger T. F. Farmer T. A. Qian T. (2013). Rapid expectation adaptation during syntactic comprehension. PLoS One 8, 1–18. doi: 10.1371/journal.pone.0077661 PMC3813674 24204909

[ref24] Foote R. Qasem M. Trentman E. (2020). Morphological decomposition in L2 Arabic: a masked priming study. J. Psycholinguist. Res. 49, 291–317. doi: 10.1007/s10936-020-09688-6, PMID: 32008140

[ref25] Gambi C. (2021). “The role of prediction in second language vocabulary learning” in Prediction in second language processing and learning. eds. Kaan E. Grüter T. (Amsterdam: John Benjamins), 187–206.

[ref26] Gambi C. Lelonkiewicz J. R. Crepaldi D. (2022). Do children (and adults) benefit from a prediction error boost in one-shot word learning? OSF. doi: 10.31219/osf.io/kx4sf PMC10785960 38223230

[ref27] Gambi C. Pickering M. J. Rabagliati H. (2021). Prediction error boosts retention of novel words in adults but not in children. Cognition 211:4650. doi: 10.1016/j.cognition.2021.104650, PMID: 33721717

[ref28] Gruber M. J. Ranganath C. (2019). How curiosity enhances Hippocampus-dependent memory: the prediction, appraisal, curiosity, and exploration (PACE) framework. Trends Cogn. Sci. 23, 1014–1025. doi: 10.1016/j.tics.2019.10.003 31706791 PMC6891259

[ref29] Grüter T. Zhu Y. A. Jackson C. N. (2021). “Forcing prediction increases priming and adaptation in second language production” in Prediction in second language processing and learning. eds. Kaan E. Grüter T. (Amsterdam: John Benjamins), 208–231.

[ref30] Hartsuiker R. Bernolet S. (2015). The development of shared syntax in second language learning. Biling. Lang. Congn. 20, 219–234. doi: 10.1017/s1366728915000164

[ref31] Hartsuiker R. Bernolet S. (2017). “Syntactic representations in late learners of a second language: a learning trajectory” in Bilingual cognition and language: The state of the science across its subfields. eds. Miller D. Bayram F. Rothman J. Serratrice L. (Amsterdam: John Benjamins), 205–224.

[ref32] Hartsuiker R. Bernolet S. Schoonbaert S. Speybroeck S. Vanderelst D. (2008). Syntactic priming persists while the lexical boost decays: evidence from written and spoken dialogue. J. Mem. Lang. 58, 214–238. doi: 10.1016/j.jml.2007.07.003

[ref33] Heyselaar E. Wheeldon L. Segaert K. (2021). Structural priming is supported by different components of nondeclarative memory: evidence from priming across the lifespan. J. Exp. Psychol. Learn. Mem. Cogn. 47, 820–837. doi: 10.1037/xlm0000955, PMID: 33151717

[ref34] Hopp H. (2016). Learning (not) to predict: grammatical gender processing in second language acquisition. Second. Lang. Res. 32, 277–307. doi: 10.1177/0267658315624960, PMID: 33507779

[ref35] Huettig F. Audring J. Jackendoff R. (2022). A parallel architecture perspective on pre-activation and prediction in language processing. Cognition 224:105050. doi: 10.1016/j.cognition.2022.105050, PMID: 35398592

[ref36] Ito A. Corley M. Pickering M. J. (2018). A cognitive load delays predictive eye movements similarly during L1 and L2 comprehension. Biling. Lang. Congn. 21, 251–264. doi: 10.1017/S1366728917000050

[ref37] Ito A. Pickering M. J. (2021). “Automaticity and prediction in non-native language comprehension” in Prediction in second language processing and learning. eds. Kaan E. Grüter T. (Amsterdam: John Benjamins Publishing), 26–46.

[ref38] Jackson C. N. Hopp H. (2020). Prediction error and implicit learning in L1 and L2 syntactic priming. Int. J. Biling. 24, 895–911. doi: 10.1177/136700692090285

[ref39] Jackson C. N. Ruf H. T. (2017). The priming of word order in second language German. Appl. Psycholinguist. 38, 315–345. doi: 10.1017/S0142716416000205, PMID: 25709590

[ref40] Jackson C. N. Ruf H. T. (2018). The importance of prime repetition among intermediate-level second language learners. Stud. Second. Lang. Acquis. 40, 677–692. doi: 10.1017/S0272263117000365

[ref41] Jaeger T. F. Snider N. (2008). “Implicit learning and syntactic persistence: Surprisal and cumulativity” in Proceedings of the 30th annual conference of the cognitive science society. eds. Love B. C. McRae K. Sloutsky V. M., 827–812. Austin, TX: Cognitive Science Society.

[ref42] Jaeger T. F. Snider N. E. (2013). Alignment as a consequence of expectation adaptation: syntactic priming is affected by the prime’s prediction error given both prior and recent experience. Cognition 127, 57–83. doi: 10.1016/j.cognition.2012.10.013, PMID: 23354056 PMC7313543

[ref43] Kaan E. Chun E. (2017). Priming and adaptation in native speakers and second-language learners. Biling. Lang. Congn. 21, 228–242. doi: 10.1017/S1366728916001231

[ref44] Kaan E. Chun E. (2018). “Syntactic adaptation” in Psychology of learning and motivation (pp. 85–116). eds. Federmeier K. D. Watson D. G. (Cambridge, CA: Academic Press)

[ref45] Kaan E. Futch C. Fuertes R. F. Mujcinovic S. de la Fuente E. Á. (2019). Adaptation to syntactic structures in native and nonnative sentence comprehension. Appl. Psycholinguist. 40, 3–27. doi: 10.1017/S0142716418000437

[ref46] Kaan E. Grüter T. (2021). “Prediction in second language processing and learning: advances and directions” in Prediction in second language processing and learning. eds. Kaan E. Grüter T. (Amsterdam: John Benjamins), 1–24.

[ref47] Kaschak M. P. Kutta T. J. Jones J. L. (2011). Structural priming as implicit learning: cumulative priming effects and individual differences. Psychon. Bull. Rev. 18, 1133–1139. doi: 10.3758/s13423-011-0157-y, PMID: 21913001 PMC4612612

[ref48] Koller M. (2016). Robustlmm: an R package for robust estimation of linear mixed-effects models. J. Stat. Softw. 75, 1–24. doi: 10.18637/jss.v075.i06 32655332 PMC7351245

[ref49] Kuperberg G. R. Jaeger T. F. (2016). What do we mean by prediction in language comprehension? Lang. Cogn. Neurosci. 31, 32–59. doi: 10.1080/23273798.2015.1102299, PMID: 27135040 PMC4850025

[ref50] Mahowald K. James A. Futrell R. Gibson E. (2016). A meta-analysis of syntactic priming in language production. J. Mem. Lang. 91, 5–27. doi: 10.1016/j.jml.2016.03.009

[ref51] Mansournia M. A. Geroldinger A. Greenland S. Heinze G. (2018). Separation in logistic regression: causes, consequences, and control. Am. J. Epidemiol. 187, 864–870. doi: 10.1093/aje/kwx299, PMID: 29020135

[ref52] Marian V. Blumenfeld H. K. Kaushanskaya M. (2007). The language experience and proficiency questionnaire (LEAP-Q): assessing language profiles in bilinguals and Multilinguals. J. Speech Lang. Hear. Res. 50, 940–967. doi: 10.1044/1092-4388(2007/067), PMID: 17675598

[ref53] Marian V. Blumenfeld H. K. Kaushanskaya M. (2020). The LEAP-Q language experience and proficiency questionnaire: ten years later. Biling. Lang. Congn. 23, 945–950. doi: 10.1017/S1366728919000038 PMC7899192 33628083

[ref54] Mitsugi S. Macwhinney B. (2016). The use of case marking for predictive processing in second language Japanese. Biling. Lang. Congn. 19, 19–35. doi: 10.1017/S1366728914000881

[ref55] Mohamed A. A. (2016). Task-based incidental vocabulary learning in L2 Arabic: the role of proficiency and task performance. J. Natl. Council Less Commonly Taught Lang. 18, 121–157. doi: 10.10117/issn.1930-9031.2015.03.08

[ref56] Montero-Melis G. Jaeger T. F. (2020). Changing expectations mediate adaptation in L2 production. Biling. Lang. Congn. 23, 602–617. doi: 10.1017/S1366728919000506

[ref57] Murayama K. (2022). A reward-learning framework of knowledge acquisition: an integrated account of curiosity, interest, and intrinsic–extrinsic rewards. Psychol. Rev. 129, 175–198. doi: 10.1037/rev0000349, PMID: 35099213

[ref58] Muylle M. Bernolet S. Hartsuiker R. (2021). The role of L1 and L2 frequency in cross-linguistic structural priming: an artificial language learning study. Biling. Lang. Congn. 24, 767–778. doi: 10.1017/S1366728921000110

[ref59] Perdomo M. Kaan E. (2019). Prosodic cues in second-language speech processing: a visual world eye-tracking study. Second. Lang. Res. 37, 349–375. doi: 10.1177/0267658319879196

[ref60] Peter M. Chang F. Pine J. M. Blything R. Rowland C. F. (2015). When and how do children develop knowledge of verb argument structure? Evidence from verb bias effects in a structural priming task. J. Mem. Lang. 81, 1–15. doi: 10.1016/j.jml.2014.12.002

[ref61] Pickering M. J. Branigan H. P. (1998). The representation of verbs: evidence from syntactic priming in language production. J. Mem. Lang. 39, 633–651. doi: 10.1006/jmla.1998.2592, PMID: 34751067

[ref62] Pickering M. J. Ferreira V. S. (2008). Structural priming: a critical review. Psychol. Bull. 134, 427–459. doi: 10.1037/0033-2909.134.3.427, PMID: 18444704 PMC2657366

[ref63] Pickering M. J. Gambi C. (2018). Predicting while comprehending language: a theory and review. Psychol. Bull. 144, 1002–1044. doi: 10.1037/bul0000158, PMID: 29952584

[ref64] Plonsky L. Oswald F. L. (2014). How big is big? Interpreting effect sizes in L2 research. Lang. Learn. 64, 878–912. doi: 10.1111/lang.12079

[ref65] Potts R. Davies G. Shanks D. R. (2019). The benefit of generating errors during learning: what is the locus of the effect? J. Exp. Psychol. Learn. Mem. Cogn. 45, 1023–1041. doi: 10.1037/xlm0000637, PMID: 30024254

[ref66] Potts R. Shanks D. R. (2014). The benefit of generating errors during learning. J. Exp. Psychol. Gen. 143, 644–667. doi: 10.1037/a0033194, PMID: 23815457

[ref67] Rabagliati H. Gambi C. Pickering M. J. (2016). Learning to predict or predicting to learn? Lang. Cogn. Neurosci. 31, 94–105. doi: 10.1080/23273798.2015.1077979, PMID: 37264936

[ref68] Reitter D. Keller F. Moore J. D. (2011). A computational cognitive model of syntactic priming. Cogn. Sci. 35, 587–637. doi: 10.1111/j.1551-6709.2010.01165.x, PMID: 21564266

[ref69] Reuter T. Borovsky A. Lew-Williams C. (2019). Predict and redirect: prediction errors support children’s word learning. Dev. Psychol. 55, 1656–1665. doi: 10.1037/dev0000754, PMID: 31094555 PMC6876992

[ref70] Ruf H. T. (2011). An investigation of syntactic priming among German speakers at varying proficiency levels. Unpublished PhD thesis. University of Wisconsin-Madison, Madison, WI.

[ref71] Sadeghi K. Sağlık E. Mede E. Samur Y. Comert Z. (2022). The effects of implementing gamified instruction on vocabulary gain and motivation among language learners. Heliyon 8:e11811. doi: 10.1016/j.heliyon.2022.e11811, PMID: 36458316 PMC9706691

[ref72] Segaert K. Wheeldon L. Hagoort P. (2016). Unifying structural priming effects on syntactic choices and timing of sentence generation. J. Mem. Lang. 91, 59–80. doi: 10.1016/j.jml.2016.03.011

[ref73] Shin J. A. Christianson K. (2012). Structural priming and second language learning. Lang. Learn. 62, 931–964. doi: 10.1111/j.1467-9922.2011.00657.x, PMID: 35389705

[ref74] van Lieshout L. L. Vandenbroucke A. R. Müller N. C. Cools R. de Lange F. P. (2018). Induction and relief of curiosity elicit parietal and frontal activity. J. Neurosci. 38, 2579–2588. doi: 10.1523/JNEUROSCI.2816-17.2018, PMID: 29439166 PMC6705901

[ref75] Wang X. Boers F. Warren P. (2022). Prompting language learners to guess the meaning of idioms: do wrong guesses linger? Lang. Aware. 31, 1–16. doi: 10.1080/09658416.2022.2153859

[ref76] Yang Y. C. Karmol A. M. Stocco A. (2021). Core cognitive mechanisms underlying syntactic priming: a comparison of three alternative models. Front. Psychol. 12:662345. doi: 10.3389/fpsyg.2021.662345, PMID: 34262508 PMC8273879

[ref77] Zhang C. Bernolet S. Hartsuiker R. (2020). The role of explicit memory in syntactic persistence: effects of lexical cueing and load on sentence memory and sentence production. PLoS One 15:e0240909. doi: 10.1371/journal.pone.0240909, PMID: 33151975 PMC7643978

[ref78] Zhang S. Hasim Z. (2022). Gamification in EFL/ESL instruction: a systematic review of empirical research. Front. Psychol. 13:1030790. doi: 10.3389/fpsyg.2022.1030790, PMID: 36687912 PMC9849815

