# Supplementary material for: What is the next structure? Guessing enhances L2 syntactic learning in a syntactic priming task
Source: Front Psychol. 2023 Jun 29;14:1188344. doi: 10.3389/fpsyg.2023.1188344 (PMC10344450; doi:10.3389/fpsyg.2023.1188344)
Supplement: Supplementary file 1 [file Data_Sheet_1.docx]

**Supplementary materials**

# Figures


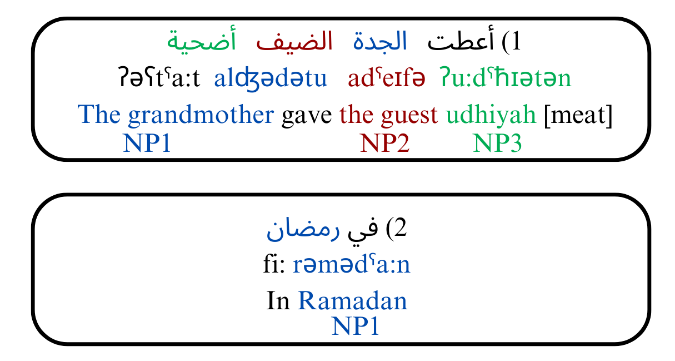


Figure 1. Number of NP arguments in the dative structure (Example 1) and TPs (Example 2).


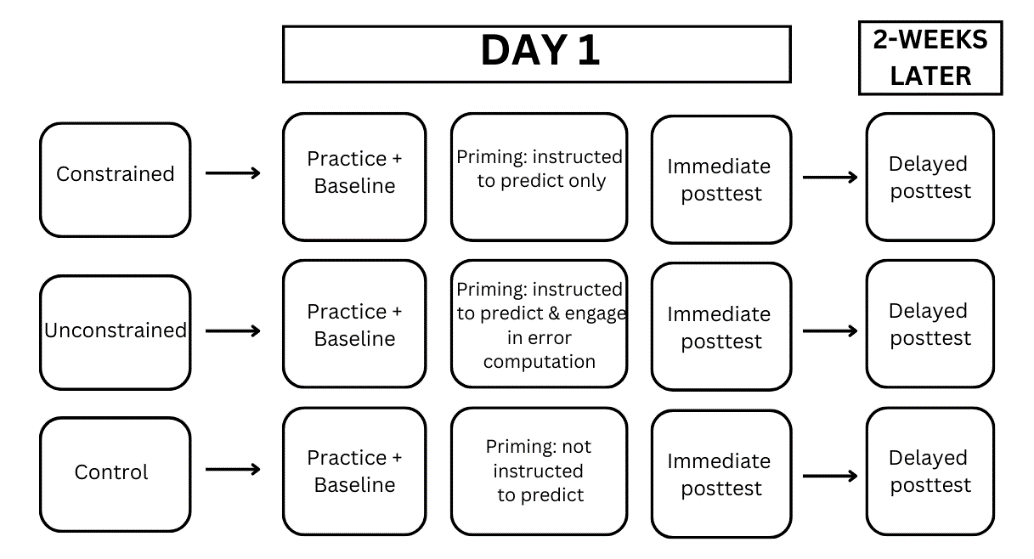


Figure 2 Caption: Flow of the priming phases across experiments by condition

Figure 2 Alt Text: Three identical experiment timelines show that three conditions completed three phases in one day and the last phase was two weeks later.


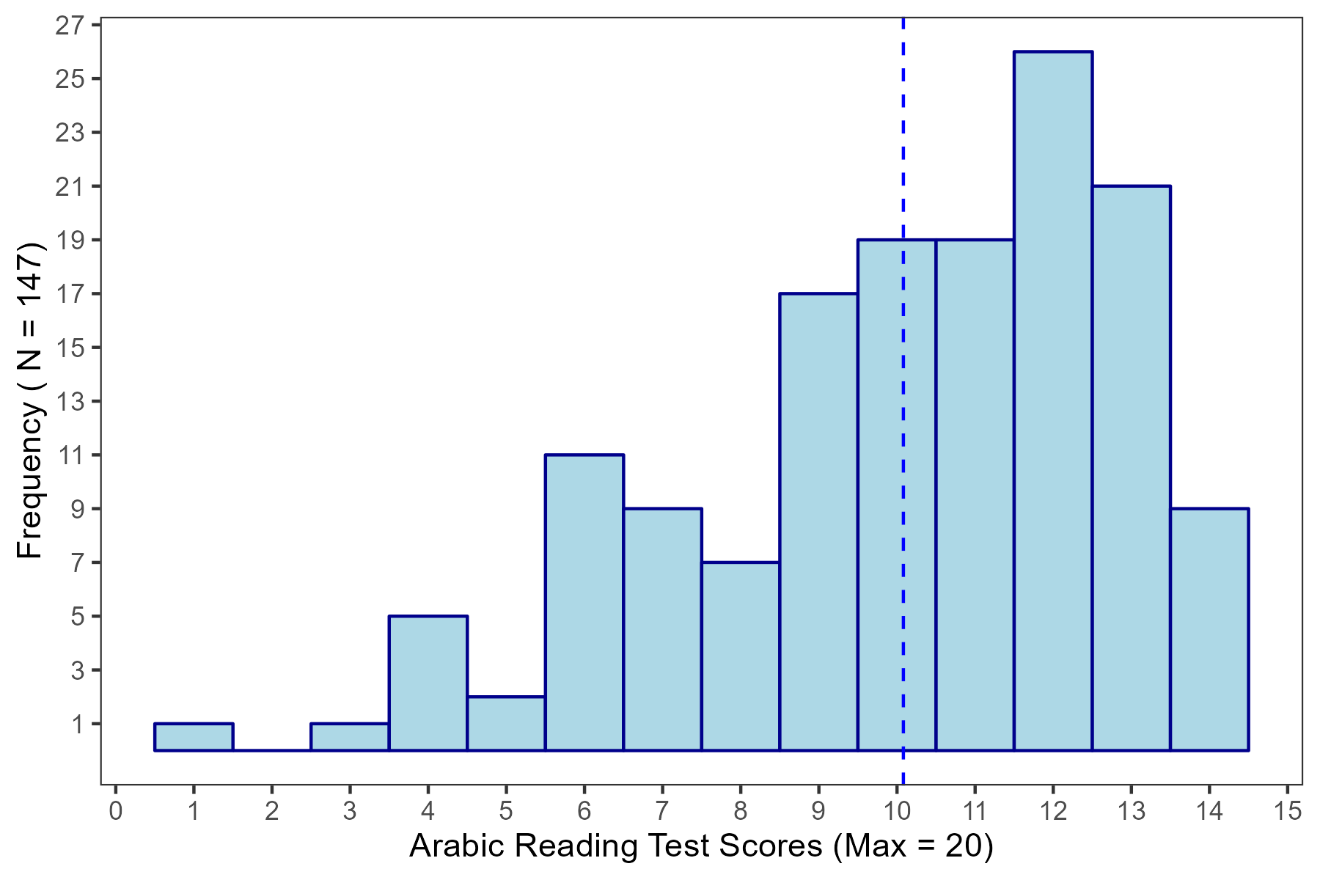


Figure 3. Distribution of the participants’ reading test scores

# Tables

Table 1. Participants demographics grouped by condition, showing mean scores, standard deviations, ranges (in parentheses), and between-group comparisons. MCQ = multiple choice question.

|  | Control  (*N* = 51) | Constrained (*N* = 46) | Unconstrained (*N* = 50) | Between-group comparisons |
| --- | --- | --- | --- | --- |
| Age | 25.1 (*SD* = 3.6) (19-35) | 25.3 (*SD* = 3.4) (20-36) | 25.6 (*SD* = 3.7) (19-35) | *χ*^2^ = .656  *p* = .72 |
| Age of first exposure to Arabic | 13.6 (*SD* = 5.6) (4-24) | 13.3 (*SD* = 6.0) (2-27) | 13.2 (*SD* = 6.2) (4-24) | *χ*^2^ = .228  *p* = .89 |
| Length of stay in Arabic-speaking countries (in years) | 5.0 (*SD* = 5.2) (0-22) | 3.8 (*SD* = 3.5) (0.1-20) | 3.6 (*SD* = 3.9) (0-20) | *χ*^2^ = 2.22  *p* = .32 |
| Length of stay in Arabic-speaking school/work environments (in years) | 5.4 (*SD* = 5.2) (0-20) | 5.5 (*SD* = 4.6) (0-23) | 5.2 (*SD* = 4.3) (0-20) | *χ*^2^ = .316  *p* = .85 |
| Self-reported Arabic proficiency (0-10) | 7.4 (*SD* = 1.9) (2.3-9.7) | 7.2 (*SD* = 1.9) (1.7-9.3) | 7.0 (*SD* = 2.1) (1.3-10) | *χ*^2^ = .943  *p* = .62 |
| MCQ Arabic reading test score (/20) | 10.1 (*SD* = 2.5) (4-14) | 10.1 (*SD* = 2.8) (3-14) | 9.9 (*SD* = 2.9) (1-14) | *χ*^2^ = .097  *p* = .95 |

Table 2. Sentence examples for the target MSA structures and their alternations

| Structure | Alternation | |
| --- | --- | --- |
| Dative | DO | PO |
|  | أعطت الجدة الضيف أضحية  ʔәʕtˤa:t alʤәdәtu adˤeɪfә ʔu:dˤћɪətәn  The grandmother gave the guest udhiyah [meat]. | أعطت الجدة أضحية للضيف  ʔәʕtˤa:t alʤәdәtu ʔu:dˤћɪətәn lidˤeɪfi  The grandmother gave udhiyah [meat] to the guest. |
| TP | Non-Fronted TP | Fronted TP |
|  | خرج الأب في الصباح  xәrәʤә alʔәbu fi: asˤәbaћi  The father went out in the morning. | في الصباح، خرج الأب  fi: asˤәbaћi xәrәʤә alʔәbu  In the morning, the father went out. |
